# Bibliometric and visual analysis of single-cell multiomics in neurodegenerative disease arrest studies

**DOI:** 10.3389/fneur.2024.1450663

**Published:** 2024-10-08

**Authors:** Jieyan Wang, Shuqing Wang, Qingyu Li, Fei Liu, Yantong Wan, Hui Liang

**Affiliations:** ^1^Department of Urology, People’s Hospital of Longhua, Shenzhen, China; ^2^First Clinical Medical School, Southern Medical University, Guangzhou, China; ^3^Guangdong Provincial Key Laboratory of Proteomics, Department of Pathophysiology, School of Basic Medical Sciences, Southern Medical University, Guangzhou, China

**Keywords:** neurodegenerative disease, single-cell RNA sequencing, cell heterogeneity, microglia, neuroscience

## Abstract

**Background:**

Neurodegenerative diseases are progressive disorders that severely diminish the quality of life of patients. However, research on neurodegenerative diseases needs to be refined and deepened. Single-cell polyomics is a technique for obtaining transcriptomic, proteomic, and other information from a single cell. In recent years, the heat of single-cell multiomics as an emerging research tool for brain science has gradually increased. Therefore, the aim of this study was to analyze the current status and trends of studies related to the application of single-cell multiomics in neurodegenerative diseases through bibliometrics.

**Result:**

A total of 596 publications were included in the bibliometric analysis. Between 2015 and 2022, the number of publications increased annually, with the total number of citations increasing significantly, exhibiting the fastest rate of growth between 2019 and 2022. The country/region collaboration map shows that the United States has the most publications and cumulative citations, and that China and the United States have the most collaborations. The institutions that produced the greatest number of articles were Harvard Medical School, Skupin, Alexander, and Wiendl. Among the authors, Heinz had the highest output. Mathys, H accumulated the most citations and was the authoritative author in the field. The journal Nature Communications has published the most literature in this field. A keyword analysis reveals that neurodegenerative diseases and lesions (e.g., Alzheimer’s disease, amyloid beta) are the core and foundation of the field. Conversely, single-cell multiomics related research (e.g., single-cell RNA sequencing, bioinformatics) and brain nerve cells (e.g., microglia, astrocytes, neural stem cells) are the hot frontiers of this specialty. Among the references, the article “Single-cell transcriptomic analysis of Alzheimer’s disease” is the most frequently cited (1,146 citations), and the article “Cell types in the mouse cortex and hippocampus revealed by single-cell RNA-seq” was the most cited article in the field.

**Conclusion:**

The objective of this study is to employ bibliometric methods to visualize studies related to single-cell multiomics in neurodegenerative diseases. This will enable us to summarize the current state of research and to reveal key trends and emerging hotspots in the field.

## 1 Introduction

In recent years, the incidence of neurodegenerative diseases has continued to increase globally with age, and is now one of the leading causes of death (1, 2). Neurodegenerative disease is a brain dysfunction caused by the loss of neurons and/or their myelin sheaths which includes both acute and chronic, and this article focuses on chronic neurodegenerative diseases, including Alzheimer’s, Parkinson’s, frontotemporal dementia, and multiple sclerosis. Patients often have lesions of hallmark amyloid deposits in the brain (3), and this pathologic change in the brain is often progressive and irreversible (4). As a result of the direct or indirect effects of altered protein conformation in the brain, patients often experience neurological abnormalities such as cognitive deficits and ataxia, which in turn affects the normal functioning of the patient’s other organ systems (5, 6). Another pathological feature of neurodegenerative diseases is neuronal inflammation, a complex process that involves the lesion and influence between various types of nerve cells, such as microglia and astrocytes (7, 8). In different regions of the brain, neurons will be selectively lost (9). The mechanism may be related to the protein conformational changes mentioned above or excitability downstream of neurotransmitter signaling and so on (10, 11). Therefore, in studies targeting the pathogenesis of neurodegenerative disorders, the application of single-cell multiomics can detect the single-cell multiomics features of neurons that are specifically sensitive to neurodegenerative disease and obtain the specific information of the affected neuronal subtypes, which will provide an invaluable reference for future basic and translational research on cellular heterogeneity in neurodegenerative disease (7, 12). Single-cell multiomics technology is a cellular sequencing method, including single-cell transcriptomics, proteomics, genomics and so on, whose research has increasing popularity in the field of neurodegenerative disease in recent years (13). Through statistics and sequencing of cytogenetic data, methods such as cellular mapping and genealogical analysis are utilized at the cellular level to perform disease studies with greater accuracy (14). Notably, single-cell sequencing is now playing an important role in the analysis of molecular subtypes of susceptible neurons in neurodegenerative diseases as well as the identification of novel mechanisms and therapeutic targets (15). It has also identified changes in the expression of disease-specific genes in certain diseases (16), such as amyotrophic lateral sclerosis which are advancing the course of clinical treatments (17). For the study of various diseases in the field of neuroscience, it is indispensable to explore the lesions at the cellular level. Thus, single-cell genomics has the obvious advantage of precise measurements, so the choice of single-cell sequencing method can undoubtedly complement the existing shortcomings of the research, especially in the context of the current increasing sophistication of single-cell multiomics technologies (18, 19).

The concern in neurodegenerative diseases continues to rise, therefore, there is a need to do a visual data statistical analysis, also known as bibliometric analysis, of existing studies on neurodegenerative diseases using single-cell sequencing. Bibliometric analysis is a method of assessing scholarly outcomes using statistics to quantitatively analyze information from purposeful literature, which includes but not limited to citations, journals, authors, and research institutions (20). The value of bibliometric methods is demonstrated in medical research (21). By fully utilizing the efficacy of bibliometric analysis, we can gain a more comprehensive understanding of the trends in the field of purposeful research (22). In this study, we used CiteSpace and VOSviewer to count the studies on the association between neurodegenerative diseases and single-cell genomics in recent years, aiming to analyze the research trends and hotspots of the related studies through bibliometric methods, and the overall analysis obtained can also be used as a reference for related researchers.

## 2 Methods

### 2.1 Data sources

Web of Science is a powerful citation search database founded in 1985 which contains a large number of interdisciplinary high-quality literature, providing a platform for researchers around the world to retrieve academic information (23). The search formula chosen for this study was set to TS = (“Single-cell transcriptome” OR “Single-cell RNA-seq” OR “Single-cell transcriptomic” OR “single-cell transcriptomics” OR “Single-Cell RNA sequencing” OR “single-cell multiomics sequencing” OR “Single-cell multiomics” OR “single-cell multiomics” OR “Single-cell genome” OR “Single-cell epigenome” OR “Single-cell epitranscriptome” OR “Single-cell proteome” OR “Single-cell metabolome” OR “scRNA-seq”) AND TS = (Alzheimer’s Disease OR Parkinson’s Disease OR Frontotemporal Dementia OR Multiple Sclerosis OR Neurodegenerative Disease OR Neurodegenerative Disorder OR Neuro-degenerative disease OR Neuro-Degenerative Disorder OR Huntington’s Disease OR Amyotrophic Lateral Sclerosis OR Alzheimers Disease OR Parkinsons Disease OR Huntingtons Disease). We searched all relevant literature between 2015 and 2024, limiting the type of literature to ARTICLE and REVIEW and the language to English during the screening process, thus excluding other types or languages, and retrieved a total of 596 articles. For a more in-depth study of single-cell sequencing, we manually screened a total of 267 articles in the field of ND that were sequenced by the authors themselves and their specific sequencing methods. To prevent data bias due to database updates, all articles in this study were collected and downloaded on June 8, 2024.

### 2.2 Data analysis and visualization

CiteSpace is a citation visualization and analysis software. Through proper use of CiteSpace, it can assist in the processing of article information and obtain an all-round and in-depth view of the cutting-edge hotspots, key information, potential trends and dynamic changes in a specific research field (24). Under the technical premise of scientometrics and data visualization, CiteSpace can also provide researchers with a diversified perspective for revealing the different fields of citation crossover, knowledge intersection and so on, providing diversified perspectives (25). CiteSpace focuses on the knowledge mapping analysis of literature to reveal the development history and research trends of literature, which helps researchers to have a more in-depth understanding of the past foundations of the research and the emerging hotspots (26). VOSviewer is a JAVA-based literature tracking software for all kinds of web data or literature data for visual analysis. VOSviewer can analyze different indicators of the literature such as the number of citations to the literature, the frequency of keywords, etc. one by one, and generate visual relationship diagrams to help researchers assess the impact of the literature and facilitate their intuitive understanding and analysis. VOSviewer focuses more on cluster analysis of the cited literature, showing the network of relationships between the literature, and is also capable of constructing a collaborative network of authors or institutions, which helps the researcher to understand the competitive or collaborative relationships in the process of research scholarship (27). In our study, we used CiteSpace to analyze reference bursts, keyword bursts, citation authors, journal bi-graph overlays, and reference collaborations and distributions. Meanwhile, we use VOSviewer to analyze country/region and institutional distribution, author distribution and collaboration, and keyword distribution and collaboration.

## 3 Results

### 3.1 Annual publications and citation trends

**Figure 1:**
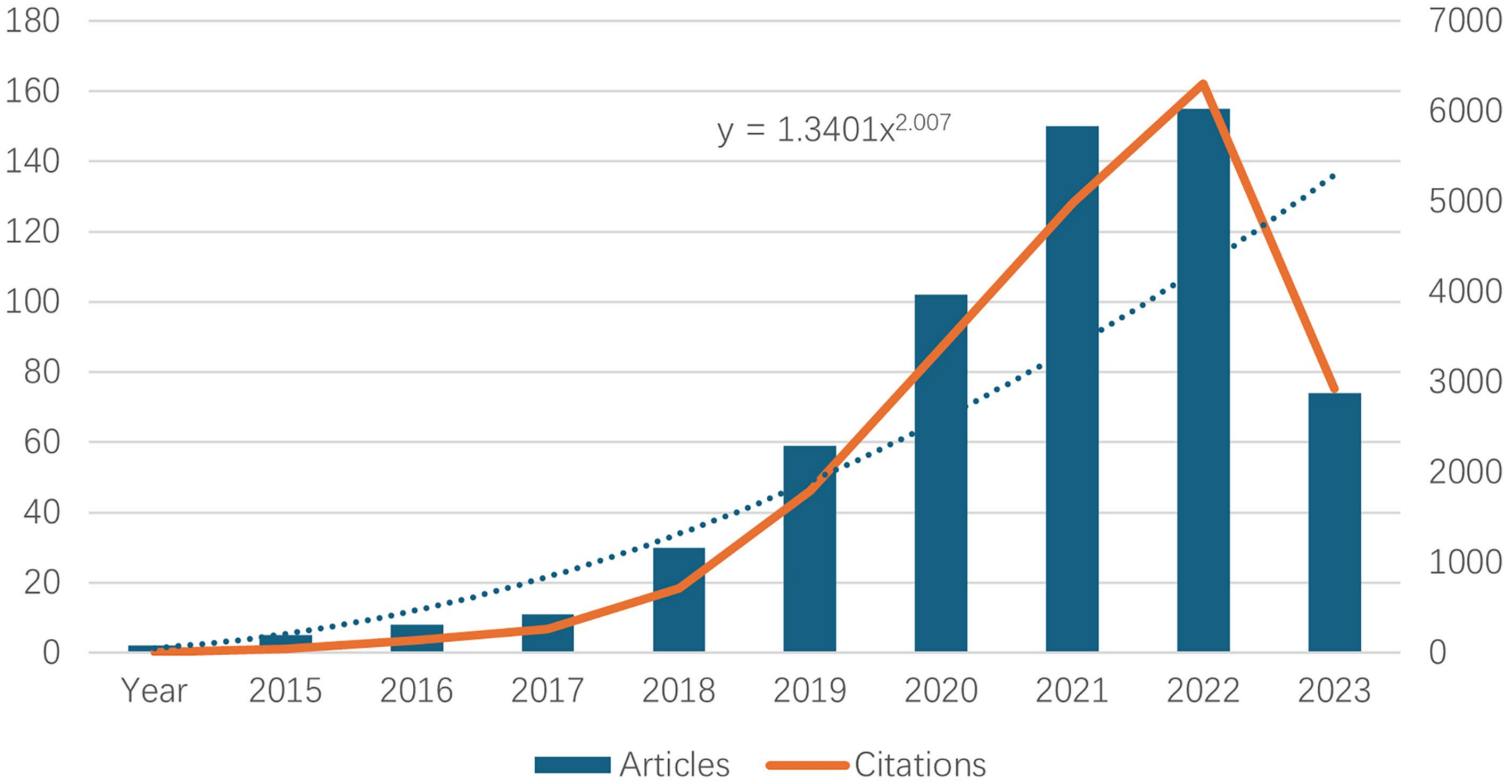
Top 10 annual neurodegenerative disease-related publications and number of related citations.

The number of literature publications can reflect directly the scientific activity and process development in a given field. Similarly, the trend in the number of citations can be used to indicate the increase in academic communication. Figure 1 show the number of publications and their corresponding citation trends from 2015 to 2024. It can be seen that the first literature on neurodegenerative disease was published in 2015. Between 2015 and 2023, the number of literature publications about neurodegenerative disease increased annually. The growth rate accelerated significantly between 2019 and 2022, with the highest number of literature publications about neurodegenerative disease in 2023, which was as high as 155. The data for 2024 were not yet available at the time of writing, but it is anticipated that the number of literature publications will continue to increase at a substantial rate, in line with the growth trend observed in Table 1. Concurrently, the number of citations has also risen significantly, indicating that research on neurodegenerative disease is gradually maturing and developing.

**Table 1:** Top 10 countries/regions in terms of number of articles published and number of citations accordingly.

Rank	Countries	Documents	Countries	Total link strength	Countries	Citations
1	USA	303	USA	207	USA	13,676
2	China	141	Germany	123	England	4,612
3	Germany	82	England	97	Germany	3,997
4	England	53	China	64	China	2,006
5	Canada	47	Netherlands	53	Sweden	1,932
6	Sweden	30	Switzerland	50	Canada	1,831
7	Switzerland	24	Canada	47	Singapore	1,343
8	Italy	21	France	45	Switzerland	1,206
9	Japan	19	Sweden	43	Netherlands	1,062
10	Netherlands	19	Denmark	38	Israel	959

### 3.2 Distribution of country/region

The geographical nature of literature publication reflects to some extent the contribution of the country or region to the field. Currently, country/regional independent or collaborative research takes place mainly in the Northern Hemisphere. Table 1 shows the top 10 countries/regions, along with their total connection strength and citation frequency. The country/regional distribution of our publications is based on the units of all authors. As can be seen from the table, the country with the highest number of articles published in the field is the United States (303), followed by China (141), Germany (82), and the United Kingdom (53). For citations to the literature, the US has the highest cumulative number of citations (13,676), followed by the UK (4,612), Germany (3,997) and China (2,006), with the cumulative number of citations in the US being almost three times that of the UK. In terms of overall connection strength, the US remains in first place, followed closely by Germany, the UK and China. In all three evaluation dimensions, the United States ranked first, and the role and status it plays in this field is evident. This may be due to the fact that there are many research institutes, associations, and universities in the U.S. that are well funded in the field of ND. At the same time, the United States has accumulated a large number of publications because of the early start of research in ND (28). Meanwhile, the number of publications from the UK is only 53, yet the number of citations is as high as 4,612, indicating that the overall quality of the literature published in the UK in this field is high and the recognition of the academic achievements is high.

**Figure 2:**
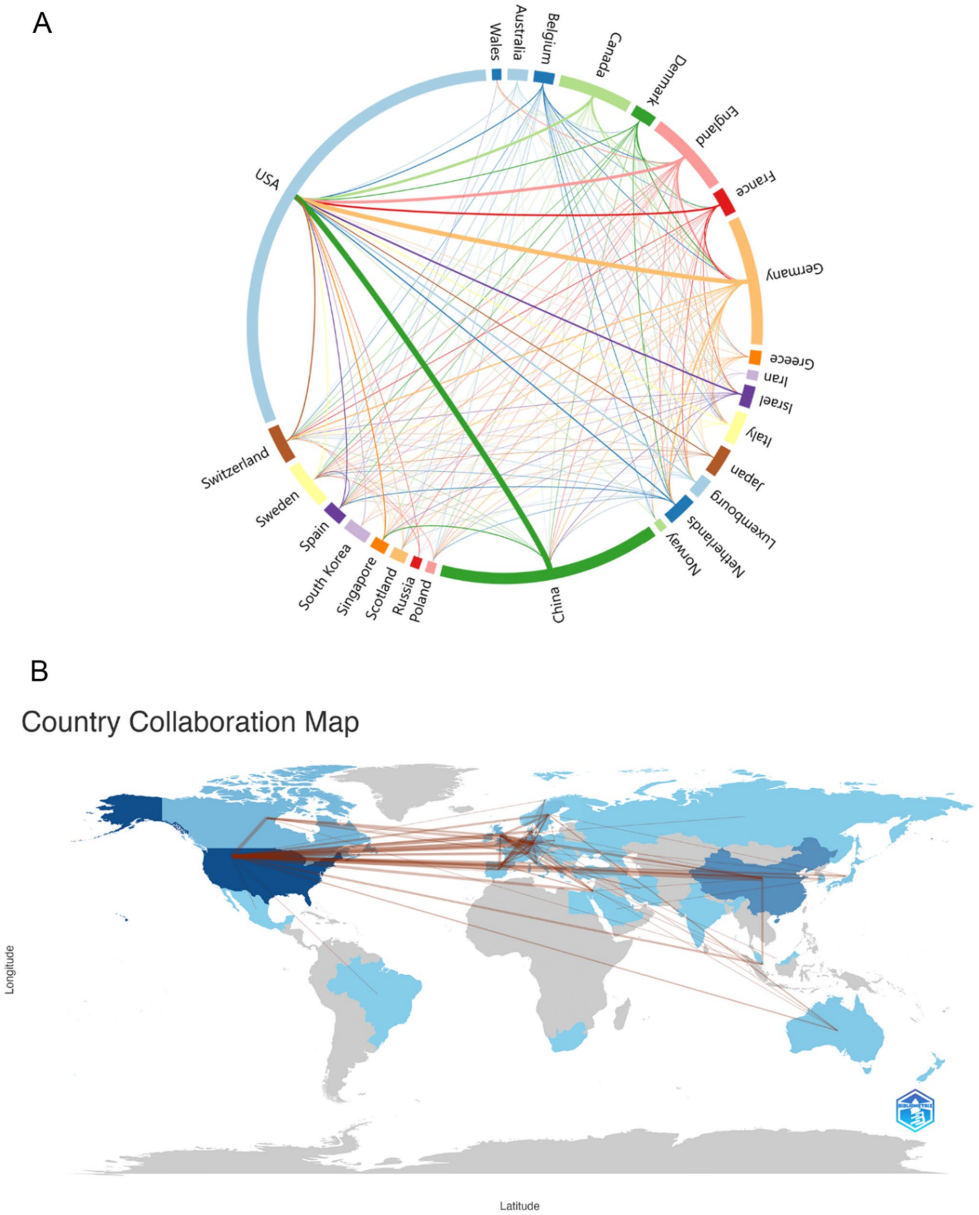
**(A)** Country/regions that have cooperation. The thickness of the lines reflects the frequency of cooperation, with thicker lines indicating more frequent cooperation. **(B)** Map of the geographic location of global collaboration. The darker the blue color in the map, the more the country/region collaborates with other countries/regions.

As can be seen from Figure 2A, we used Charticulator to perform the data processing and visual analysis, and counted the countries/regions with more than 5 publications in this field and visualized the frequency of their collaborations. Figure 2A shows the cooperation between countries/regions in the field of using single-cell omics to study neurodegenerative disease with the United States and China cooperating most closely, followed by the United States and Germany. Countries/regions with collaborative relationships can support each other in this field of research, promote each other’s academic research process and maximize research efficiency. Figure 2B presents a map of the cooperation between countries and regions. It can be observed that North America, Europe, and East Asia in northern hemisphere are the most active in this field while Russia, Saudi Arabia and India are also involved in research in this direction. In conjunction with Figure 2A, it can be discerned that China and the United States are in frequent contact with Europe.

### 3.3 Distribution by institutions

Table 2 shows the top 10 institutions in terms of number of publications and citations related to single-cell multiomics in the neurodegenerative disease research area. The institution with the highest number of publications was Havard Med Sch (29), followed by Broad Inst MIT & Havard (23), Univ Calif San Diego (23), and Univ Calif San Francisco (22). In terms of the number of citations in the literature, the institution with the highest number of citations was Broad Inst MIT & Havard (4,175), followed by Havard Med Sch (3,018) and MIT (2,568). Most of the top 10 institutions are from the United States, which means that the United States has a significant presence and contribution in this field. The leading status of Havard Med Sch and Broad Inst MIT & Havard among these agencies suggests that these two agencies play a significant role in this regard.

**Table 2:** Top 10 institutions in neurodegenerative disease related to single cell omics.

Rank	Institution	Publications	Original country	Institution	Citations	Original country
1	Harvard Med Sch	29	United States	Broad Inst MIT & Harvard	4,175	United States
2	Broad Inst MIT & Harvard	23	United States	Harvard Med Sch	3,018	United States
3	Univ Calif San Diego	23	United States	MIT	2,568	United States
4	Univ Calif San Francisco	22	United States	Univ Cambridge	1,890	United States
5	Washington Univ	21	United States	Univ Freiburg	1,738	Germany
6	Karolinska Inst	20	Sweden	Karolinska Inst	1,716	Sweden
7	Johns Hopkins Univ	19	United States	Rush Univ	1,701	United States
8	Icahn Sch Med Mt. Sinai	17	United States	Stanford Univ	1,355	United States
9	Mcgill Univ	16	Canada	Tech Univ Munich	1,354	Germany
10	Stanford Univ	15	United States	Univ Calif San Francisco	1,343	United States

**Figure 3:**
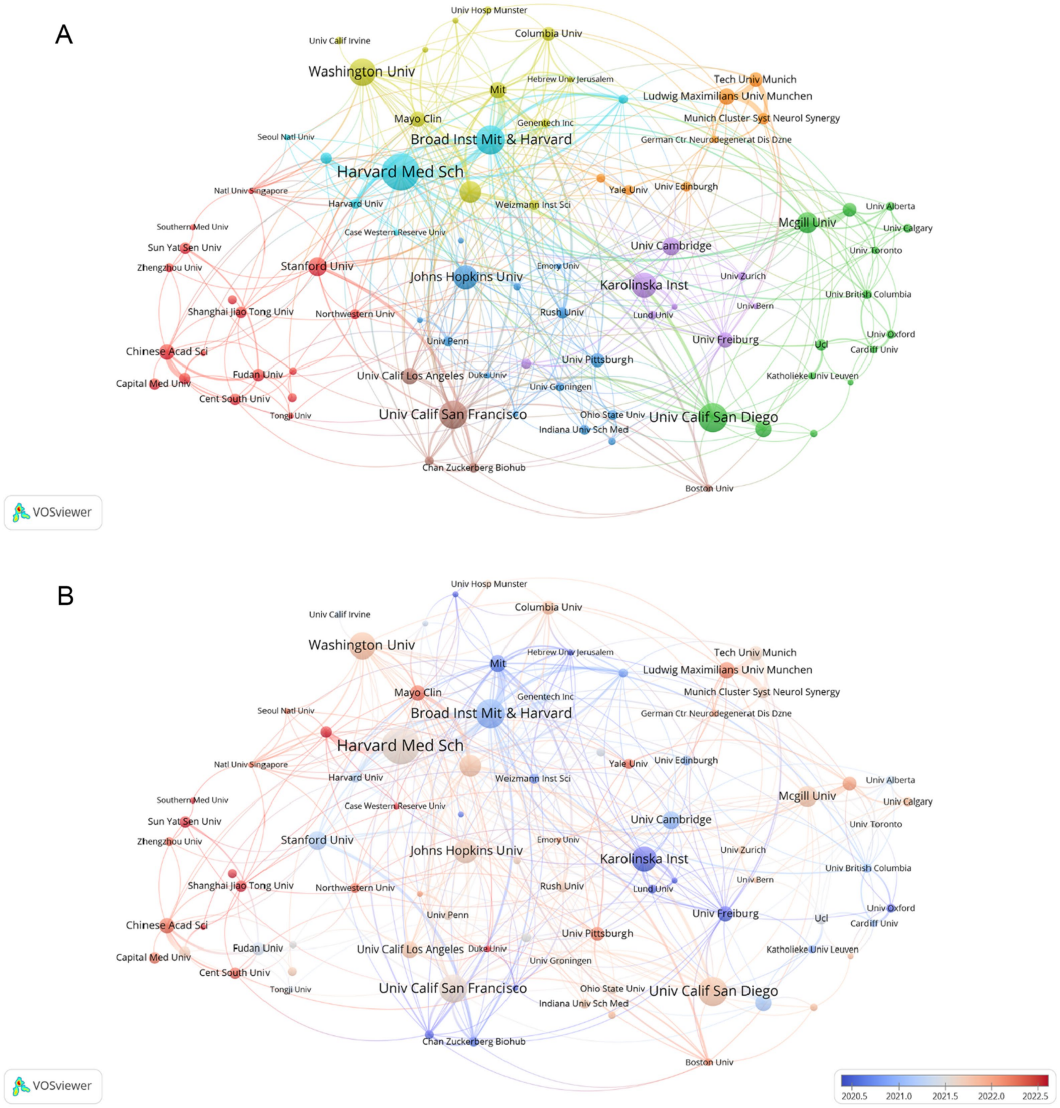
**(A)** Analysis of collaborative network visualization of different institutions in VOSviewer. The figure shows the collaborative institutions with more than 1 number of documents. The nodes of different colors represent the collaborative relationships between institutions with different clusters, and the size of the nodes indicates their number of publications. **(B)** The average publication time of the institution is indicated by the color of the node. Blue indicates an earlier average publication time, while red indicates a later average publication time. The size of the node is proportional to the number of articles published by the partner institution.

Figure 3A, generated by VOSviewer, screens for collaborating institutions with more than 5 publications, revealing the distribution of institutional collaborations in studies related to single-cell multiomics in neurodegenerative disease. The collaborative relationships between institutions are grouped into eight tightly knit clusters, which are differentiated by color in the figure. The network of lines formed by Broad Inst MIT & Havard and Havard Med Sch can be seen to be very densely distributed and occupies a central position in the cooperation map, reflecting the close cooperation and influence of the two. Furthermore, the Stanford University, Chinese Academy of Sciences, and other institutions in the red group, as well as the Johns Hopkins University, University of Pittsburgh, and other institutions in the blue group, engage in close collaboration with the Karolinska Institute and the University of Cambridge, which are included in the purple group. Figure 3B, generated by VOSviewer, shows the average posting time for organizations from 2020.5 to 2022.5. Institutional nodes such as Havard Med Sch & Washington Univ are mainly indicated by light red color, indicating relatively new involvement in this direction but a high number of publications. Some institutions such as Broad Inst MIT & Havard and Karolinska Inst have larger blue nodes, indicating that they have been involved in the field earlier to conduct research. The remaining organizations with smaller nodes with red color, such as Shanghai Jiao Tong Univ and Sun Yat Sen Univ, participated in the study later and published little literature, but may play a leading role in future studies.

### 3.4 Distribution of authors

A co-cited author is a co-cited relationship between two authors when a third author cites both authors. A higher frequency of common citations indicates a closer academic interest and research density (29). Table 3 shows the number of publications, co-citations, affiliation, corresponding country/region, and total link strength for the top 10 authors. Most published authors are Skupin, Alexander (Luxembourg Institute of Health) are Wiendl, Heinz (Universitätsklinikum Münster), both of which have published seven articles. It shows that these two authors have contributed more in this domain. The author with the highest total number of co-citations regarding this line of research is Mathys, H from University of Pittsburgh, with a cumulative total of 185 citations and a total connection strength of 2,654. It is noteworthy that half of the authors who ranked in the top 10 in respect of co-citations in this field were from the United States, reflecting the larger impact and academic credibility of the United States in using single-cell multiomics to study neurodegenerative disease.

**Table 3:** Top 10 authors and co-cited authors in neurodegenerative disease related to single-cell multiomics.

Rank	Author	Documents	Countries/regions	Institution	Author	Co-citations	Countries/regions	Institution
1	Skupin, Alexander	7	Luxembourg	Luxembourg Institute of Health	Mathys, H	185	United States	University of Pittsburgh
2	Wiendl, Heinz	7	Germany	Universitätsklinikum Münster	Keren-Shaul, H	149	Israel	Weizmann Institute of Science
3	Amit, Ido	6	Israel	Weizmann Institute of Science Israel	Stuart, T	145	United Kingdom	Cranfield University
4	Colonna, Marco	6	United States	Washington University	Butler, A	105	United Kingdom	University of Sussex
5	Grzyb, Kamil	6	Luxembourg	University of Luxembourg	Masuda, T	97	Japan	Universidad de Bienestar Médico de Kawasaki
6	Parmar, Malin	6	Sweden	Lund University	Grubman, A	94	United States	Tufts University School of Medicine
7	Prat, Alexandre	6	Canada	Université de Montréal	Zeisel, A	85	United States	University of North Carolina at Chapel Hill
8	Prinz, Marco	6	Germany	University Hospital Freiburg	Zhou, YY	85	United States	University of California, San Diego
9	Zu Horste, Gerd Meyer	6	Münster	University of Münster,	Zhang, Y	79	China	Xiamen University
10	Antel, Jack	5	Canada	Montreal Neurological Institute	Hammond, TR	73	United States	Harvard Medical School

**Figure 4:**
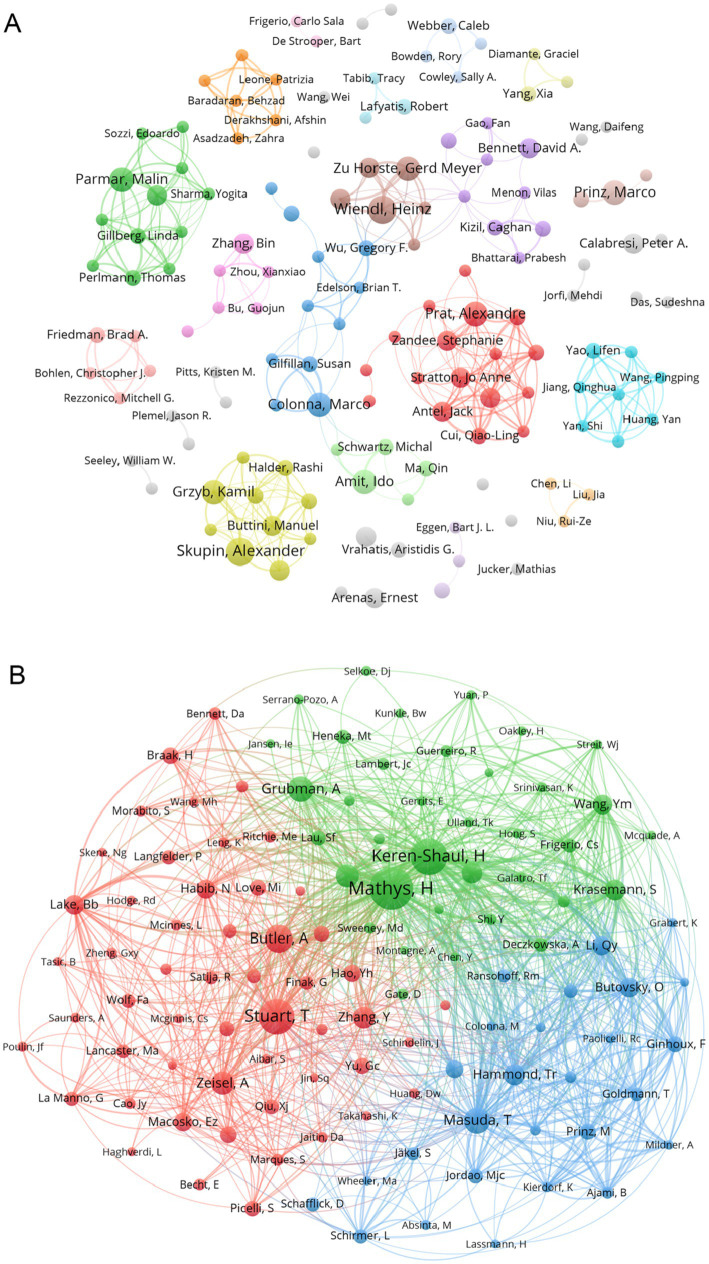
**(A)** Collaborative network of authors associated with single-cell multiomics in ND. Different colour clusters represent different author collaborations. **(B)** Co-cited authors in neurodegenerative disease related to single-cell polyomics. Different node colors represent authors in different clusters, with larger nodes representing stronger co-authorship links or a higher number of published articles.

We screened co-authors with more than 3 publications and visualized them with VOSviewer. Figure 4A depicts the clusters of co-authors’ collaborations. The more connected group, led by Prat, Alexandre (including Antel, Jack and Zandee, Stephanie et al.), is represented by red, which symbolizes their more frequent collaborations. Next came the yellow-green group (including Skupin, Alexander, and Grzyb et al.) and the green group (including Parmar, Malin, and Gillberg, Linda et al.) In addition, other cluster authors have some collaborations, such as the brown group (including Zu Horste, Gerd Meyer and Wiendl, Heinz et al.) and the light blue group (including Yao, Lifen and Wang Pingping et al.). Figure 4B represents the number of documents produced by co-authors, again, Prat, Alexandre and his co-authors have the larger red nodes, indicating that Prat, Alexandre and Zandee, Stephanie et al. are the core force in this research area and play an significant role in the current research process. In addition, two clusters of co-cited authors, Skupin, Alexander and Colonna, Marco, have published more articles. We also analysed the co-cited-authors of articles in the ND field that were sequenced by the authors themselves, which are pictured in Supplementary Figure S8. By comparing Supplementary Figure S8 with Figure 4B, we found an emerging cluster (in yellow) that consists of Keren-Shaul H and his other co-authors. It indicates that Keren-Shaul H, Wang, Ym, and Krasemann, S et al. have made more use of single-cell sequencing methods in ND research, and have provided helpful databases for single-cell studies in ND. Based on the timeline plot of the authors’ outputs (Supplementary Figure S3) we can see that Amit, Ido and Zhang, Bin published their articles earlier, suggesting that they were early adopters of single-cell multiomics approaches in the field of neurodegenerative disease research. Meanwhile, all authors on the 2021 graph have published relevant literature, indicating that a trend toward applying single-cell multiomics to neurodegenerative disease research arose during this period. As illustrated in the graph of the distribution of corresponding authors by country (Supplementary Figure S2), China and the United States continue to occupy the top positions, with the majority of them exhibiting a greater number of transnational co-authorships than domestic co-authorships. Germany, Canada, the United Kingdom and Sweden followed.

### 3.5 Distribution of journal

**Figure 5:**
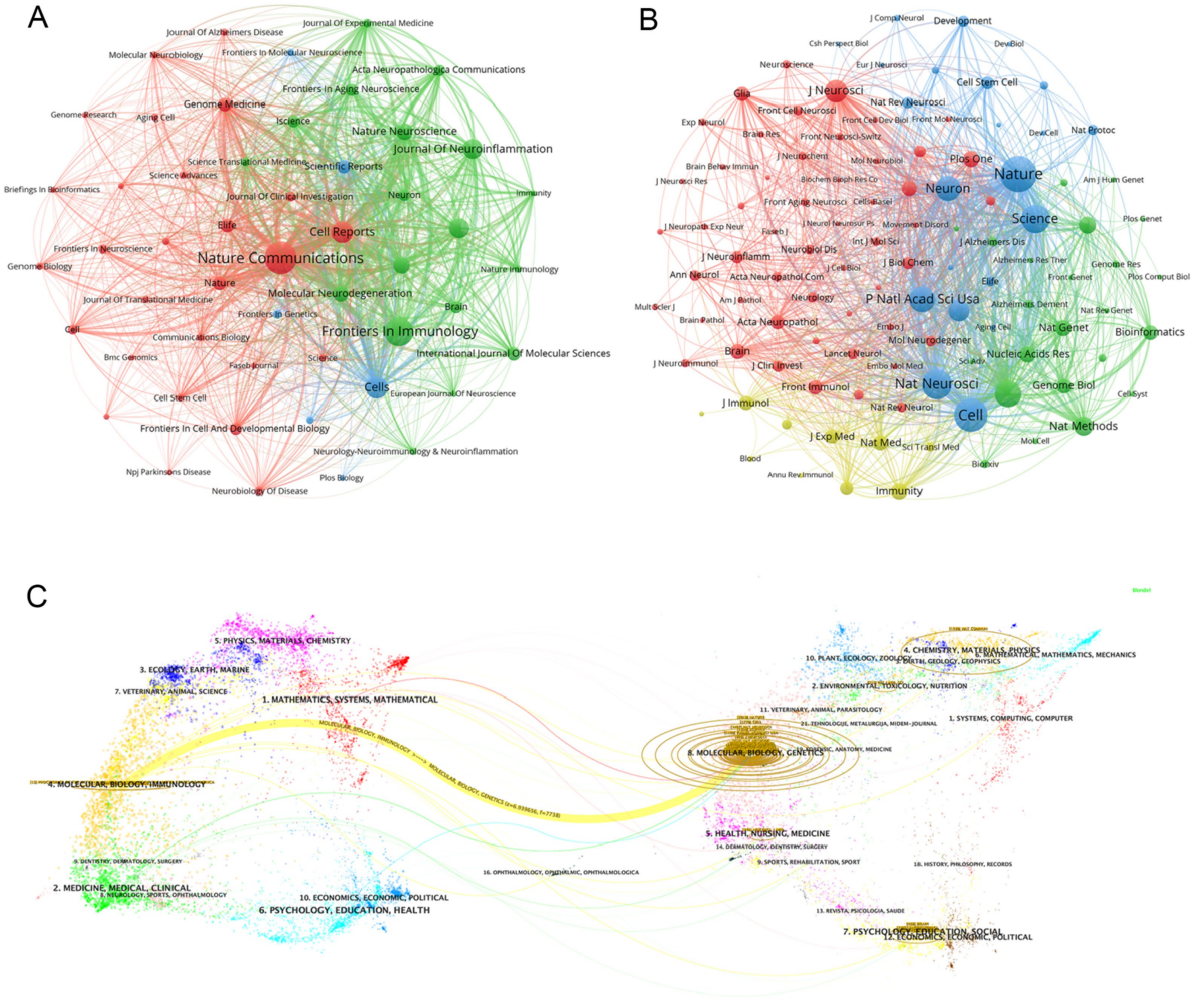
**(A)** Graph of the number of literature related to single-cell multiomics in journal publication neurodegenerative disease The size of the node area corresponds to the number of journal releases. Different clusters are distinguished by color. **(B)** Journal collaboration clustering graph. Different colored nodes indicate different clusters of collaborating journals. **(C)** The dual-map overlay of journals publishing articles related to single-cell multiomics in neurodegenerative disease. The width of the connecting lines represents the strength of the citation relationship, with thicker lines indicating stronger citation relationships.

We identified a total of 596 articles on Alzheimer’s disease microglia published in 201 journals. Through the application of VOSviewer, we visualized the number of journal publications and clustering collaboration. As shown in Table 4, the journal with the highest number of relating publications was Nature Communications (29), followed by Frontiers in Immunology (27), Cell Reports (17) and Cells (17). Among the top 10 journals by number of publications, five journals have an IF value of 10 or more. Among the top 10 journals in terms of co-citation frequency, eight journals have an IF value of 10 or more. The most cited journals were Nature (2,021) and Cell (1,770), reflecting the overall high quality of research in the field in these journals. The top 10 journals in terms of publications and the top 10 journals with regard to citations are all from the Q1 division, indicating that journals from the Q1 division are more inclined to be regarded as reliable sources of citations and have a more influential impact. Figure 5A presents the results of the clustering analysis of journals. It is evident that the clustering network diagram of journals is centered on Nature Communications. The journals in the red group are the primary sources of literature in the field, including Nature Communications, Cell Reports, and others. The green group comprises journals that are also highly regarded, such as Frontiers in Immunology and the Journal of Neuroinflammation. The blue group includes journals such as Cells and Scientific Reports, which are also considered to be of significant importance. Among all these groups, red and green groups take center stage. These journals that dominate the field have a higher impact factor and greater authority in their own right. In the field of neuroscience, the Journal of Neuroinflammation is a specialised journal and is therefore favoured by authors. In Supplementary Figure S4, we can see intuitively that the journals with more publications, such as Nature Communications, Cell Reports and Nature, etc. published the relevant literature earlier, indicating that they have made many prospective studies, laying a solid foundation for the subsequent research process. While Brain, International Journal of Molecular Science and Journal of Neuroinflammation are late in publishing relevant literature. As Table 4 and Figure 5B illustrate, the collaborating journals have been classified into four primary clusters. The red cluster encompasses journals such as J. Neuroscience, Glia, and Brain, which concentrate on the neurological aspects of the brain. The blue group encompasses a number of highly regarded scientific journals, including Nature, Neuron, Cell, and Science. These journals cover a diverse range of content and exert a considerable influence within the pharmaceutical field. The green group includes journals such as Nature Methods, Nature Genetics, and Bioinformatics, which focus on bioinformatics. The light green group Includes journals such as Immunity, J Exp Med, and J Immunol, which focus on bioimmunity-related content. Similarly, we analysed the co-cited journals for articles in the field of ND whose authors had performed single-cell sequencing (Supplementary Figure S9). We found some adjustments in the clustering of co-cited journals compared to Figure 5B. The clustering of some of the articles changed, e.g., Nat Neurosci is now grouped with journals such as Neuro and J Neruo. It may indicate that citations to journals categorised according to specialisation are more frequent in articles where single-cell sequencing has been performed.

**Table 4:** Top 10 journals in the neurodegenerative disease field concerning the number of publications and citations related to single-cell polyomics.

Rank	Journal	Publications	IF (JCR2022)	JCR quatile	Co-cited-journal	Citations	IF (JCR2022)	JCR quatile
1	Nature Communications	29	16.6	Q1	Nature	2,021	64.8	Q1
2	Frontiers In Immunology	25	7.3	Q1	Cell	1,770	64.5	Q1
3	Cell Reports	17	8.8	Q1	Nat Neurosci	1,467	25	Q1
4	Cells	17	6	Q1	Science	1,359	56.9	Q1
5	Journal OF Neuroinflammation	15	9.3	Q1	Nat Commun	1,230	16.6	Q1
6	Proceedings of the National Academy of Sciences of the United States of America	15	11.1	Q1	Proc Natl Acad Sci USA	1,160	11.1	Q1
7	Frontiers In Cellular Neuroscience	12	5.3	Q1	Neuron	1,109	16.2	Q1
8	Nature Neuroscience	12	25	Q1	J Neurosci	926	5.3	Q1
9	Molecular Neurodegeneration	11	15.1	Q1	Cell Rep	843	8.8	Q1
10	Nature	9	64.8	Q1	Nat Methods	714	48	Q1

Figure 5C shows the journal overlay diagram generated by CiteSpace, where journals citing other journals are located on the left side while the cited journals are distributed on the right side. The journal overlay graph can visualize the citation relationship between journals, citation frequency, and the disciplinary crossover between different fields (30). In the figure we observe the most obvious a yellow connecting line connecting the left and right areas, denoting that studies published in journals related to the field of Molecular, Biology, and Genetics mainly cited literature published in journals related to the field of Molecular, Biology, and Immunology, and the two are closely linked under the research direction. At the same time, we can also see that relevant literature from the fields of Medicine, Medical, Clinical, Psychology, Education, Health, and Mathematics, Systems, and Mathematical are highly cited, which means that the theoretical content of the research is closely related to the reality of clinical care. The close connection of the theoretical research content with the reality of clinical medical care further illustrates that the academic intersection of different research fields contributes to our understanding of the use of single-cell multiomics in the context of neurodegenerative disease. The core journal area is clearly visible in Supplementary Figure S5, which includes journals such as Nature Communications, Frontiers in Immunology, and Cell Reports. This is consistent with the data in Table 4. Supplementary Figure S5 graphically visualizes the comparison of journal publications, with Nature Communications and Frontiers in Immunology having about twice as many publications as the other journals.

### 3.6 The analysis of hotspots and frontiers

#### 3.6.1 Keyword cluster and timing analysis

Keywords can succinctly summarize the research focus of an article, and the analysis of keywords assists us in understanding the research focus, research trends, and emerging areas in the intended research direction. Table 5 demonstrates the top 20 keywords that appeared in the articles related to single-cell multiomics in the neurodegenerative disease study. The keywords “single-cell RNA sequencing” (171) and “Alzheimer’s disease” (120) have the highest frequency and the corresponding total link strength. Then followed by “microglia” (68) and “multiple sclerosis” (47). Alzheimer has more publications than other neurodegenerative diseases. This may be due to the fact that Alzheimer’s disease itself has a large patient base, a low cure rate, a high impact on life, and a high level of societal concern (31). At the same time, Alzheimer’s disease has the typical pathological changes of neurodegenerative diseases, and research on Alzheimer’s disease can also contribute to the research on other neurodegenerative diseases to a certain extent.

**Table 5:** Top 20 keywords with appearances related to single-cell polyomics in the neurodegenerative disease field.

Rank	Keyword	Occurrences	Total link strength	Rank	Keyword	Occurrences	Total link strength
1	single-cell RNA sequencing	171	332	11	RNA-seq	13	38
2	Alzheimer’s disease	120	261	12	machine learning	12	33
3	microglia	68	186	13	inflammation	10	30
4	multiple sclerosis	47	89	14	brain	9	33
5	Parkinson’s disease	36	71	15	cellular heterogeneity	9	20
6	neurodegenerative disease	34	84	16	experimental autoimmune encephalomyelitis	9	23
7	neuroinflammation	27	58	17	ipsc	9	21
8	astrocyte	21	59	18	bioinformatics	8	16
9	transcriptome	21	51	19	glia	8	25
10	aging	19	43	20	heterogeneity	8	18

**Figure 6:**
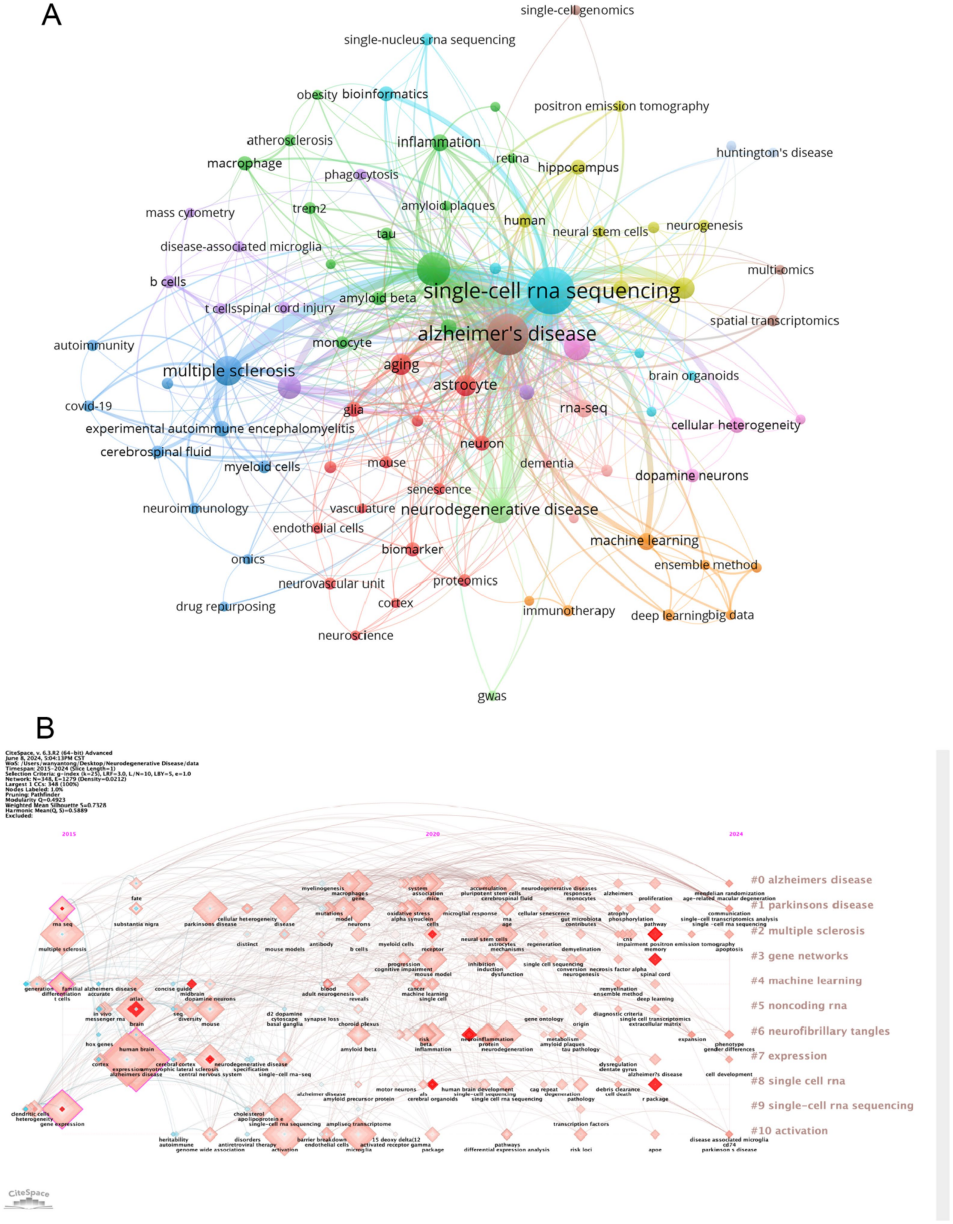
**(A)** Cluster analysis of keywords. Nodes of different colors represent keywords of different clusters. **(B)** A timeline view of the keywords. Each horizontal line represents a cluster; the smaller the number, the larger the cluster. The size of the nodes reflects the co-citation frequency, and the connecting lines between the nodes indicate the co-citation relationship of the keywords.

**Figure 7:**
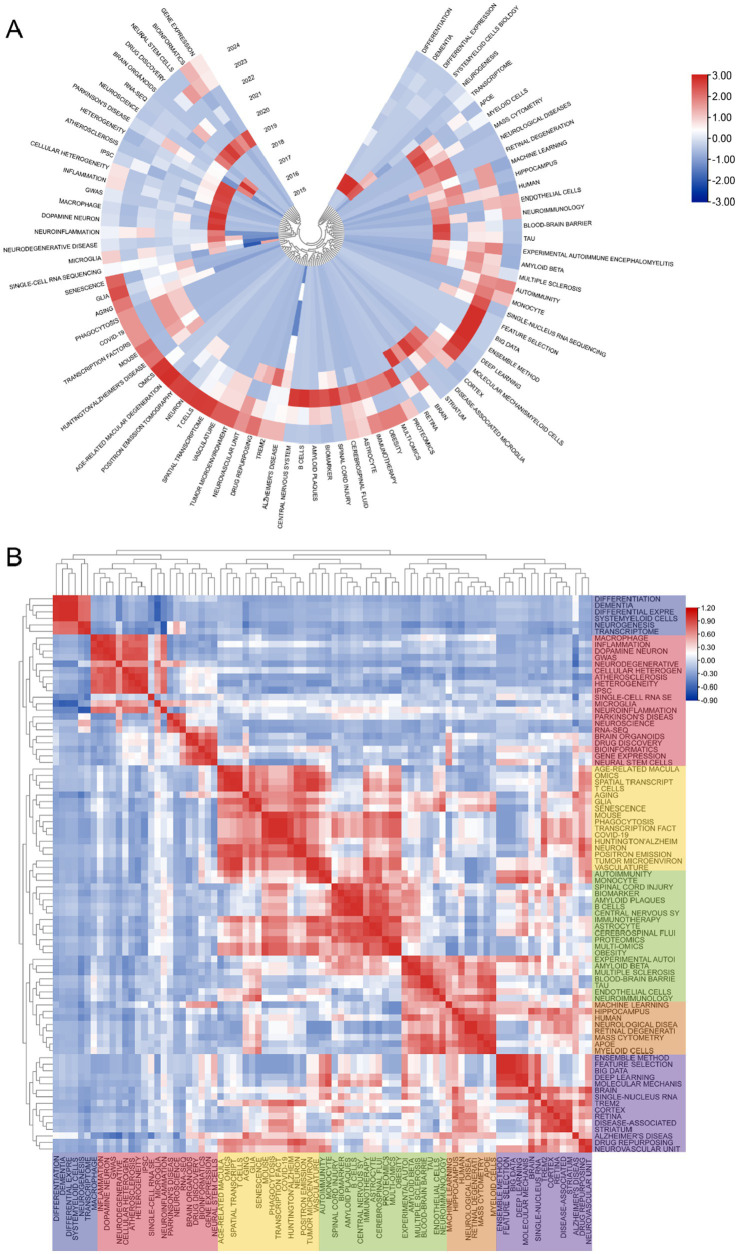
**(A)** The following graph depicts the annual keyword heatmap from 2015 to 2024. The annual heat value of each keyword is calculated by dividing the number of citations in a given year by the total number of citations that citations in the year. **(B)** Keyword relevance heatmap. Keyword with high popularity in a similar time period are grouped together, with different categories distinguished by different colors.

The cluster analysis of the keywords is shown in Figure 6A. The brown nodes are grouped to symbolize the neurodegenerative diseases themselves and the basic research methods that include “Alzheimer’s disease” “spatial transcriptomics”; the light blue nodes include the keywords of single-cell multiomics and its related researches including “single-cell RNA sequencing” “bioinformatics” etc.; red nodes include keywords related to brain cell such as “astrocyte” “glia” and so on; the dark green nodes contain keywords for pathological changes such as “amyloid beta” “inflammation” etc. The dark blue nodes contain keywords for cerebrospinal fluid components such as “multiple sclerosis” “myeloid cells” etc.; the light green nodes contain keywords for nerve cells such as “hippocampus” “myeloid cells” etc. The purple nodes include keywords related to diseased cells such as “disease-associated microglia” “B cells” and so forth. Orange nodes include keywords for data analysis methods such as “big data” “machine learning” and so forth. We also analysed keyword clustering for articles using single-cell sequencing methods. In the Supplementary Figure S10 it can be seen that the keywords of these articles are more centred on the level of the cells and their connections to each other. Supplementary Figure S6 presents a clear analysis of the average year of occurrence of each keyword with the change from blue to red indicating the temporal progression. One can see that keywords such as “ran-seq” and “macrophage” appear in 2021. Keywords such as “astrocyte” “single-cell RNA sequencing” “amyloid beta” “neuron” and “Alzheimer’s disease” appear mainly in 2022 which indicating their centrality to the research in the field. The remaining keywords such as “multiomics” “monocyte” “cerebrospinal fluid” “neuron” and “autoimmunity” appeared later which undoubtedly provide directional references for future research. This suggests that the frontier of the field may be focused on research in the direction of cerebrospinal fluid autoimmunity and so on. The average year of occurrence of each keyword in articles in the ND field where single-cell sequencing was performed can be seen in the Supplementary Figure S11. It can be seen that words such as “microglia” and “amyloid beta” appear earlier and words such as “t cells” and “memory b cells” appear later. We can see that in the visualization of the average number of citations per year for keywords (Supplementary Figure S7) several keywords with high cumulative citation counts such as “Alzheimer’s disease” “astrocyte” “neurodegenerative disease” have a high average number of citations per year representing the key part of this domain. However the terms “myeloid cells” and “deep learning” have a lower average number of citations per year indicating that research in these areas may not be as abundant. In the Supplementary Figure S12 you can see the average number of citations per year for the keywords of the articles that sequenced single cells. We found that the keywords with the higher average number of citations were “microglia,” “astrocyte” and “neuroinflammation.” It can be seen that microglia and astrocyte are popular targets for single-cell sequencing. Figure 6B also shows the timeline of the keywords. We can see that the earliest and largest cluster is #0 Alzheimer disease illustrating that the research in this field is basically centered around this central keyword. At the same time 2 out of these 10 clusters are still developing, i.e., the research direction represented by the keyword is frontier of research. The emerging keyword clusters include #3 “gene networks” and #8 “single cell RNA.” This in part reveals that gene network interactions may be an emerging hotspot for ND research while single-cell RNA sequencing is an effective tool to help probe. In the annual heat map of keyword (Figure 7A) we can see more specific keyword heat over time. Over time the keywords that received more attention changed from “differentiation” “dementia” “differential expression” and “systemyeloid cells biology” to “neuroinflammation” “dopamine neuron” “gwas” and “cellular heterogeneity” etc. In recent years terms like “blood-brain barrier” “myeloid cells” “disease-associated microglia” “feature selection” “cerebrospinal fluid” and “astrocyte” has emerged as popular concepts. It can be observed that the keyword heat represents the focal point of research and that people’s attention to neurodegenerative disease gradually shifts from superficial diseases to deep cellular and molecular mechanisms. This year’s hot keywords include “neurovascular unit” “aging” “trem2” and “glia.” Due to the incomplete statistics the results of this year’s keywords may have some deviation. Figure 7B illustrates the correlation between keywords with those that are more prevalent during a given period grouped into distinct clusters of 6 colors. This includes blue clusters (“SYSTEMYELOID CELLS” “MACROPHAGE” “DIFFERENTIAL EXPRE MACROPHAGE” “DIFFERENTIAL EXPRE” “NEUROGENESIS” etc.) the red clusters (“INFLAMMATION” “ATHEROSCLEROSIS INFLAMMATION” “ATHEROSCLEROSIS” “RNA-SEQ” and “BIOINFORMATICS” etc.) yellow clusters (“AGE-RELATED MACULA” “AGING” and “GLIA” etc.) green clusters (“AUTOIMMUNITY” “SPINAL CORD INJURY” “BIOMARKER” and “AMYLOID BETA” etc.) orange clusters (“MACHINE LEARNING” “MACHINE LEARNING” etc.) and the “MACHINE LEARNING” cluster (“MACHINE LEARNING” etc.). (“MACHINE LEARNING” “MASS CYTOMETRY” and “APOE” etc.) purple clusters (“BIG DATA” “MASS CYTOMETRY” and “AMYLOID BETA” etc.) purple clusters (“BIG DATA” “MOLECULAR MECHANIS” “TREM2” and “STRIATUM” etc.).

#### 3.6.2 New insights from single-cell sequencing

As technology advances, the use of single-cell sequencing in ND has become more diverse. A new technology that has emerged in recent years is spatial transcriptomics, and spatially resolved transcriptomics has been named “Method of the Year” for 2020 by Nature Methods (32). Due to the limitations of single-cell sequencing, single-cell multiomics alone may result in the loss of three-dimensional information about cell populations (33). Therefore, single-cell sequencing combined with spatial transcriptomics will be a future trend (34). There are now some articles on single-cell sequencing combined with spatial transcriptomics studies of ND, but the number is not large (35). Here we propose this possible hotspot in the hope that it can provide some help to future researchers studying neurodegenerative diseases.

### 3.7 References bursts and references distribution

**Figure 8:**
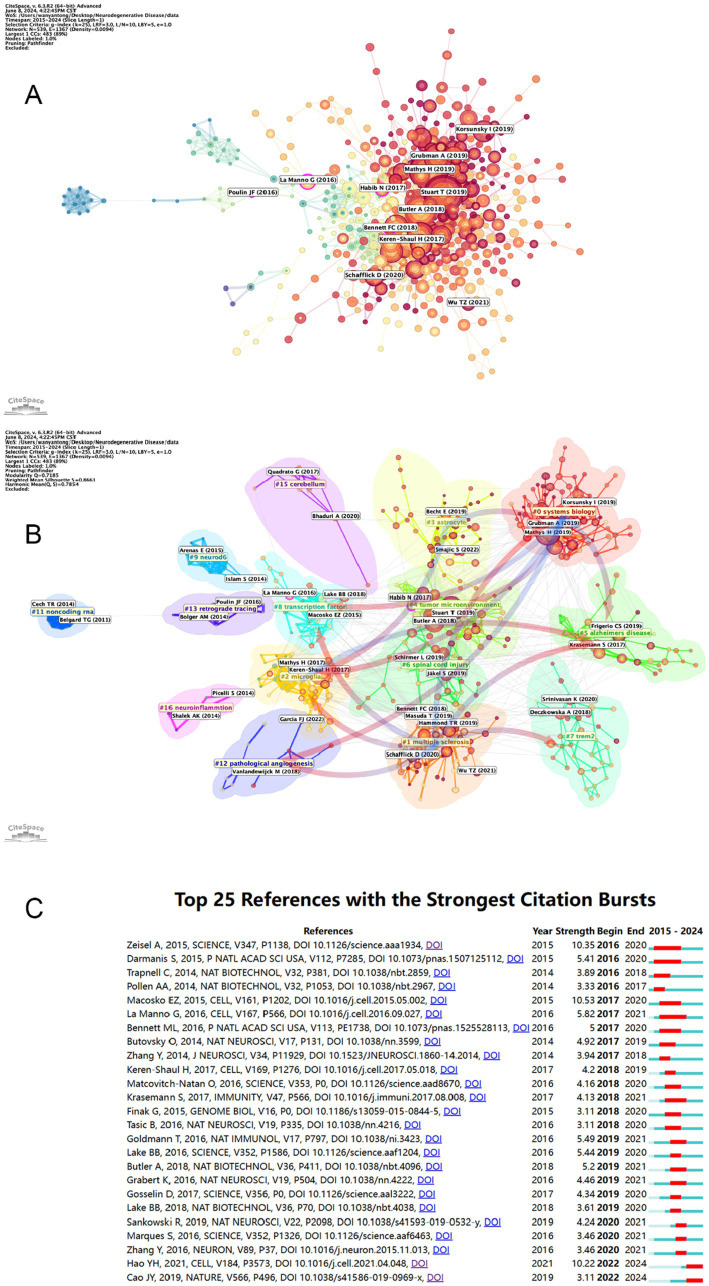
**(A)** Visual mapping of article citation counts. Nodes are represented by the first author for the corresponding article. **(B)** Visual mapping of article citation counts. Nodes in different colored regions represent different article clusters. **(C)** The top 25 citations with the strongest reference bursts and their total connection strength and publication time. The blue line marks the time interval, while the duration of the reference burst is shown by the red line.

Table 6 and Figure 8A present a visual representation of the 15 most frequently cited articles in the retrieved paper pool. The article “Single-cell transcriptomic analysis of Alzheimer’s disease” was the most frequently cited article (1,146) (19), which employed single-cell sequencing (SCS) to assess the frontal cortex of Alzheimer’s disease (AD) patients and quantify the correlation between gene expression in specific cell types and pathological trait variability. This approach revealed the heterogeneity of diseased neuronal cells in Alzheimer’s disease patients and facilitated a deeper understanding of the pathological features and pathogenesis of AD. The second most cited article is “Single-cell RNA sequencing of microglia throughout the mouse lifespan and in the injured brain reveals complex cell-state changes” (36). This article is intended to use scs to determine the different states of microglia, and in turn help to identify specific markers of microglia state that can be used to diagnose disease, providing a new advancement in the study of therapeutic targets for neurodegenerative disease “Developmental heterogeneity of microglia and brain myeloid cells revealed by deep single-cell RNA sequencing” is the third most-cited (37). In this article, researchers reveal the heterogeneity of microglia, and complement the current gene expression dataset of microglia from adult and diseased brains. “Genetic architecture of Parkinson’s disease” is the fourth most-cited (38). This study takes full advantage of single-cell multiomics to accurately measure cell specificity, summarizes the current status and progress of Parkinson’s disease research and predicts the future of Parkinson’s genetics. By analyzing the network of citation relationships of the articles, we made a visual graph of the clusters of the citation counts of the articles (Figure 8B). Cluster #0 “systems biology” is the largest cluster, indicating that the paper associated with it has been cited the most times. It can be seen that the most frequent keyword in the clustering of all articles is “systems biology,” followed by #1 “multiple sclerosis,” #2 “microglia” and #3 “astrocyte,” indicating that brain nerve cells are the major research focus and supporting basic theories in this field. The arrows in the figure indicate the evolution of the article clusters. The earliest research in the field is represented by the following clusters: #1 “multiple scleriosis,” #9 “neurod6,” #13 “retrograde tracing,” #15 “cerebellum,” and #16 “neuroinflammation.” These clusters are independent of each other. Subsequently, cluster #1 evolved into #0 “systems biology,” #2 “microglia,” #5 “Alzheimer disease,” #7 “trem2,” #8 “transcription factor” and #12 “pathology angiogenesis,” while the evolutionary domains of #0 highly overlap with #1. It is noteworthy that both #2 and #5 represent the direction of evolution in numerous areas, thereby underscoring the significance of this cluster. Figure 8C shows the time span analysis of the burst references, with statistics collected between 2015 and 2024. The article with the strongest outburst was “Cell types in the mouse cortex and hippocampus revealed by single-cell RNA-seq” by Zeisel et al. in 2015, revealing diversity of brain cell types and transcriptomes. The articles that are still bursting are “Integrated analysis of multimodal single-cell data” and “The single-cell transcriptional landscape of mammalian organogenesis.” Both articles employed single-cell polyomics to determine cellular subtypes, thereby revealing cellular heterogeneity. It is worth mentioning that the article “The single-cell transcriptional landscape of mammalian organogenesis” broke out at a later time, but still has strong total connectivity strength, suggesting that the research direction of single-cell multiomics to measure cellular subtypes involved in this article may be a current research hotspot.

**Table 6:** Top 15 cited articles related to single-cell polyomics in the field of ND.

Rank	Author	Article title	Source title	Cited	Year	Document type	DOI
1	Mathys, H. et al.	Single-cell transcriptomic analysis of Alzheimer’s disease	Nature	1,146	2019	Article	10.1038/s41586-019-1195-2
2	Hammond, TR. et al.	Single-cell RNA sequencing of microglia throughout the mouse lifespan and in the injured brain reveals complex cell-state changes	Immunity	1,092	2019	Article	10.1016/j.immuni.2018.11.004
3	Li, QY. et al.	Developmental heterogeneity of microglia and brain myeloid cells revealed by deep single-cell RNA sequencing	Neuron	564	2019	Article	10.1016/j.neuron.2018.12.006
4	Blauwendraat, C. et al.	The genetic architecture of Parkinson’s disease	Lancet Neurology	537	2020	Review	10.1016/S1474-4422(19)30287-X
5	Jordao, MJC. et al.	Single-cell profiling identifies myeloid cell subsets with distinct fates during neuroinflammation	Science	513	2019	Article	10.1126/science.aat7554
6	Cochain, C. et al.	Single-cell RNA-seq reveals the transcriptional landscape and heterogeneity of aortic macrophages in murine atherosclerosis	Circulation Research	505	2018	Article	10.1161/CIRCRESAHA.117.312509
7	La Manno, G. et al.	Molecular diversity of midbrain development in mouse, human, and stem cells	Cell	490	2016	Article	10.1016/j.cell.2016.09.027
8	Gate, D. et al.	Clonally expanded CD8 T cells patrol the cerebrospinal fluid in Alzheimer’s disease	Nature	462	2020	Article	10.1038/s41586-019-1895-7
9	Kapellos, TS. et al.	Human monocyte subsets and phenotypes in major chronic inflammatory diseases	Frontiers in Immunology	442	2019	Review	10.3389/fimmu.2019.02035
10	Mathys, H. et al.	Temporal tracking of microglia activation in neurodegeneration at single-cell resolution	Cell Reports	433	2017	Article	10.1016/j.celrep.2017.09.039
11	Gaublomme, JT. et al.	Single-cell genomics unveils critical regulators of Th17 cell pathogenicity	Cell	416	2015	Article	10.1016/j.cell.2015.11.009
12	Xiong, XL. et al.	Landscape of intercellular crosstalk in healthy and NASH liver revealed by single-cell secretome gene analysis	Molecular Cell	412	2019	Article	10.1016/j.molcel.2019.07.028
13	Olah, M. et al.	Single cell RNA sequencing of human microglia uncovers a subset associated with Alzheimer’s disease	Nature Communications	301	2020	Article	10.1038/s41467-020-19737-2
14	Falcao, AM. et al.	Disease-specific oligodendrocyte lineage cells arise in multiple sclerosis	Nature Medicine	291	2018	Article	10.1038/s41591-018-0236-y
15	Molgora, M. et al.	TREM2 modulation remodels the tumor myeloid landscape enhancing anti-PD-1 immunotherapy	Cell	275	2020	Article	10.1016/j.cell.2020.07.013

## 4 Discussion

In this bibliometric analysis, we examined 586 research articles pertaining to single-cell histology in neurodegenerative disease studies using CiteSpace 6.3R2 Advanced, VOSviewer 1.6.18, and Charticulator. The data were retrieved on June 8, 2024 from the Web of Science Core Collection database and were utilized evaluate the spatial and temporal distribution, contributions, core articles, research hotspots, and frontiers of this field. It is essential to count articles that use two or more multiomics approaches that provide us with different research perspectives on the same organisation.

### 4.1 General information

The annual number and trends of papers may help to understand the development and progress of this study. As shown in Figure 1, from 2015 to 2018, the number of papers was within 10, with a slow growth, indicating that single-cell multi omics research on neurodegenerative diseases is still in its early stages. From 2019 to 2023, there has been a significant increase in articles related to this field, indicating that this research field is becoming increasingly important. It should be noted that over half (51.18%) of the papers have been published in the past 2 years (2022–2023), indicating that single-cell multi omics research on neurodegenerative diseases has received increasing attention from scholars in recent years.

From the distribution of countries/regions, it can be seen that the top three countries with published papers are the United States, China, and Germany. The papers published in the United States have also been cited the most, indicating its dominant position in this field. In addition, among the top 10 institutions that publish papers, 8 are from the United States; Among the top 10 institutions in terms of citation frequency, 7 also come from the United States. These findings indicate that the United States plays a decisive role in this field.

Table 4 reveals that Nature Communications has published the most papers on single-cell omics research on neurodegenerative diseases and ranks fifth among the most cited journals. Among the top 10 journals with the most published papers, 7 of them also ranked among the top 10 with common citations (Nature Communications, Cell Reports, Cells, Proceedings of the National Academy of Sciences of the United States of America, Nature Neuroscience, Nature, Journal of Neuroinflammation), which reveals their high academic value in this field. Furthermore, it is worth mentioning that the top 10 journals with the highest number of published papers are Q1 or Q2, indicating that the quality of publications in this field is generally high. Among the top 10 journals, 4 mainly involve the field of neuroscience, while other journals are related to biochemistry, molecular biology, cell biology, and interdisciplinary fields (by number of publications).

According to Figure 4B, the green clustering is represented by Keren Shaul, H, and Mathys, H, which focus on using single-cell omics to analyze cell heterogeneity in neurodegenerative diseases. The main articles cited in this clustering are “Single cell transcriptomic analysis of Alzheimer’s disease” and “A Unique Microglia Type Associated with Restricting Development of Alzheimer’s Disease,” which fully utilize single-cell omics for cell heterogeneity analysis in the exploration of neurodegenerative diseases and have achieved significant results. The blue clustering, represented by Hammond and TR, focuses on the research of the occurrence and development of various neurological diseases, focusing on the role of neural cells, especially microglia, in neurodegenerative diseases. Red clustering, represented by Stuart and T, tends to favor single-cell omics analysis methods and techniques. It should be pointed out that the scholar’s paper titled “Comprehensive Integration of Single Cell Data” presents a strategy for the assembly of harmonized references and transfer of information across datasets, enabling the researchers to integrate single-cell measurements not only across scRNA-seq technologies, but also across different modalities, and has significant influence in this field (39).

### 4.2 Effects of cellular communication between different cells on neurodegenerative disease

Keywords such as “glia,” “neural stem cells” and “T cells” represent the forefront of single-cell multiomics research in neurodegenerative diseases (ND), with numerous studies in this field related to intercellular communication (40). Single-cell multiomics can play a significant role in the study of intercellular communication, which undoubtedly holds an important position in the disease process of ND (41). Here, we explore intercellular communication in ND research based on different cell types. T cells are a crucial component of the human immune system, and studies have indicated that an increase in T cell numbers is associated with tau pathology, highlighting their significant role in ND (42). A study has pointed out that in Alzheimer’s disease (AD), there is extensive inferred communication between CD4^+^ T cells and CD8^+^ T cells. CD4^+^ T cells appear to facilitate the recruitment and adhesion of CD8^+^ T cells, potentially promoting T cell infiltration into the brain parenchyma (43). In Parkinson’s disease, research has found that CD4 cytotoxic T lymphocytes (CTLs) are recruited by SPP1 and secrete the cytokine IFNG, thereby activating endothelial cells (ECs), disrupting the blood-brain barrier (BBB), promoting the recruitment of immune cells, and ultimately advancing the progression of Parkinson’s disease (PD). Wang’s et al. (40) research found that the number and strength of outgoing communications from CD8 TEMRA cells, which communicate with CD8 TEM and CD4 TEM, are downregulated in AD patients, indicating weakened intercellular communication. This leads to a corresponding weakening of control by other immune cells such as CD4 TEM and CD8 TEM over CD8 TEMRA cells, thereby exacerbating the development of AD. Subsequent studies have further confirmed that the immune mechanism of AD involves the clonal expansion of T cells in the cerebrospinal fluid (44). Concurrently, pathological interactions between TFH cells and B cells in the cerebrospinal fluid may also locally drive autoimmune responses in the central nervous system (45). Jorfi’s et al. (46) research revealed the role of infiltrating T cells in triggering INF/inflammatory-related pathways, including associations with microglia and IFN (e.g., IFITM1 and STAT1), antigen presentation (e.g., MHC-I and MHC-II), and pro-inflammatory cytokines or chemokines (e.g., CXCL10 and IL-32). This study also points out the potential role of the CXCL10 receptor CXCR3 in AD. Additionally, research has found that communication mediated by CXCL16-CXCR6 between microglia and CD8 T cells exists in multiple AD mouse models (47). The CXCR6-CXCL16 axis may be involved in the recruitment and maintenance of clonally expanded CD8 T cells in the cerebrospinal fluid of patients with multiple sclerosis (48). In the cerebrospinal fluid environment of individuals with cognitive impairment, myeloid cells communicate with CD8 T cells through the CXCL16-CXCR6 pathway (49). In other words, the CXCL16/CXCR6 pathway between T cells and microglia may be an important target for the treatment of other neurological diseases and neurodegenerative diseases related to neuroinflammation in the central nervous system (50).

Microglia can regulate the homeostasis of the central nervous system and play a significant role in the development of the neural environment and neuroinflammation (51). A study using sequencing identified microglial genes associated with aging with genes positively correlated with age including those controlling interferon signal transduction such as *Cxc16* and *Gas6* antigen presentation such as *H2-D1* and *H2-Q7* lipid metabolism regulation genes immune response regulation genes phagocytosis regulation genes and oxidative stress response regulation genes; negatively correlated genes include microglial marker genes chemokine signal genes ER-associated protein degradation genes and iron metabolism genes (52). Another study pointed out that the OA2 microglial cell subset promotes age-related brain inflammation by expressing some unique inflammatory signals (such as Lgals3 Cst7 Ccl4 Ccl3 Il1b) (36). Since aging is a major influencing factor of neurodegenerative diseases (53) such research not only plays a key role in elucidating the mechanisms of aging but also provides innovative methods for the treatment of neurodegenerative diseases in the future. A study pointed out that the SPP1-CD44 ligand-receptor pair in microglia has the highest contribution to ALS disease (54). Another study showed that microglia target oligodendrocytes through the APP-NGFR and CXCL12-CXCR4 axes and target OPCs through the FGL1-EGFR axis activating the apoptosis of necrotic cells and thus promoting the development of AD. In addition CXCR4 epidermal growth factor receptor MAP4K4 and IGF1R were identified as potential biomarkers and candidate therapeutic targets for the disease (55). Mifflin et al. (56) discovered a type of inflammatory microglia regulated by the RIPK1 gene RIMM. In RIMM cytokines such as TNF are upregulated promoting inflammatory pathways in ALS which can be treated with RIPK1 inhibitors. Notably Lee’s et al. (57) research revealed that increased communication between excitatory neurons L6b and microglia drives a significant upregulation of the cerebrospinal fluid pathway in AD patients which is an inflammatory pathway. The study found ligand-target links such as APOE-ABCA1 and PSEN1-APP between microglia and neurons with mutations in *PSEN1* and *PSEN2* being associated with the early onset of amyloid precursor protein in AD. In addition in the communication from astrocytes to neurons the study also found ligand-target links that connect neuro-risk genes with potential upstream effectors such as APP-TREM2 and APP-ABCA1 links.

Astrocytes are supportive cells for neurons and are also involved in the composition of the blood-brain barrier, the perception of neurotransmitters, and the nutritional delivery of neurons (58). An innovative study used the snATAC-seq method to directly compare two epigenetic data types in glial cells and found that C9-ALS-related changes in chromatin accessibility were positively correlated with *H3K27ac* in both astrocytes and microglia (59). Sun’s et al. (60) article indicated that increased communication between astrocytes and neurons in the mouse brain promotes neuronal energy metabolism. They found that the supplementation of short-chain fatty acids can significantly regulate neurotransmitter uptake and upregulate genes involved in astrocyte-neuron metabolic coupling (including *Glul, Slc1a2,* and *Gstm1*), thereby improving Alzheimer’s disease symptoms in mice. Conversely, impaired energy metabolism leads to neuronal dysfunction. Although this study cannot directly apply the results to humans, it does provide new insights and emphasizes the importance of intercellular communication in neurodegenerative diseases. Another study observed that the ligand BMP7 and receptor BMPR1A + ACVR2A, BMPR1B + ACVR2A, and BMPR1B + BMPR2 displayed close communications in normal astrocytes to OPC (61). In ALS and FTD diseases, research has found that endoplasmic reticulum (ER) stress and DNA damage response (DDR) are the main pathways affecting diseases in astrocytes and C9 ALI-COs neurons. Related genes include *DDIT3, SP1, NFE2L2,* and *EGR1* in astrocytes and *DDIT3, FOSL1, ATF3, LEF1,* and *NFATC1* in neurons (62). Siddiqui’s et al. (63) research showed that when the nerve growth factor (Ngfr) signal is induced and activated in the hippocampus of an AD pathological mouse model, it reduces the reactive glial state by inhibiting Lcn2/Slc22a17 signal transduction and enhances the neurotrophic function of astrocytes. Some astrocytes are characterized by reduced gene expression driven by NRF2 and increased signaling of MAFG and MAT2a, promoting central system inflammation in experimental autoimmune encephalomyelitis, which may lead to the pathogenesis of MS. (64) In addition, during the literature review process, the important role of extracellular vesicles (EVs) in communication between neurons was also discovered. A study found that communication between glial cells and neurons through vesicles is necessary for neuronal growth and cell survival. There is evidence that EVs are involved in the physiological interactions between all cells that make up the neurovascular unit and the intrinsic development and protection of neuronal tissue (65). Another study also focused on vesicles and neurons, finding that vesicle exocytosis abnormalities related to the development of axonal spheroids near amyloid-beta plaques, a marker of AD lesions. Vesicles containing CD63 occur and accumulate at the axonal terminals to form spherules, which can damage synaptic connections in the hippocampal-commissural pathway (66). A study using a zebrafish single-cell sequencing database to map all possible communication maps conducted through fgfr3 and its ligands (67).

The above cells are not all the cells involved in critical communication in ND, and we have discussed only a few of the most heated cells. And technological advances in single-cell multiomics have the ability to break down technical barriers and take disease research to the next level (68). In response to these specific diseases, the exchange of information between different cells forms a complex communication network that either promotes or antagonizes each other (61). This feature represents a significant challenge in the field of neurodegenerative disease therapeutic research. In conclusion, the cellular communication information obtained through the single-cell multiomics assay has advanced the study of neurodegenerative disease pathogenesis, pathogenesis, and drug targets. Moreover, this information will continue to play an important role in subsequent studies.

### 4.3 Using single-cell RNA sequencing decoding cellular heterogeneity in AD

Neurodegenerative diseases are typically characterized by delayed progressive damage to specific (sub)cell populations of the nervous system that are critical for mobility, coordination, strength, sensation and cognition. The selection and study of these specific cells has become feasible with the advent of single-cell histology techniques, which are now state-of-the-art methods capable of analyzing the heterogeneity of complex tissues, including the human post-mortem brain, at very high resolution (69). In recent years, more and more attention has been paid to exploring the cell heterogeneity in the occurrence and development of neurodegenerative diseases by using monocytomics. The research on cell heterogeneity of various cells in AD has also become a hotspot in recent years. The single cell sequencing of ND is mainly used in the research of mouse models and postmortem human brains, and mouse models are widely used to simulate the occurrence and development of diseases. However, in the research of human neurodegenerative diseases, human tissues, that is, postmortem human brains, are also essential research materials, which can provide the most practical data for disease research to build appropriate models.

Astrocytes are the most widely distributed type of cells in mammalian brain, and also the largest type of glial cells. This kind of glial cells are star shaped and send out many long and branching protrusions from the cell body, which extend and fill between the nerve cell bodies and its protrusions, playing the role of supporting and separating nerve cells. In view of its important role in brain homeostasis, the heterogeneity of this cell has attracted the attention of researchers. Mathys et al. (19) identified pathologically related glial cell types and genes through scRNA seq in AD patients: AD pathologically related OL lineage cells characterized by high expression of *CRYAB* or *QDPR, GLUL* and *CLU* are preferentially expressed in AD pathologically related astrocyte subtypes. These genes may become markers of the pathologically related glial cell subtypes in AD. It is worth noting that the heterogeneity and function of disease related reactive astrocytes are also related to a variety of neurodegenerative diseases. After that, Smith et al. (70) found that the inflammatory pathway was significantly enriched in astrocytes in the sequencing of the dead brain, and the phagocytosis, inflammation and protein inhibition pathways were enriched in microglia and perivascular macrophages, with a high content of tissue amyloid protein. The researchers also found that there are some distinguishable subsets in astrocytes and microglia. In the study of mouse models, Habib et al. (71) defined AD related astrocytes (DAA) in the hippocampus and prefrontal cortex of AD mice, and their number increased with the progress of the disease. They successfully demonstrated that DAA plays a role in the initial stage of AD by regulating the pathway related to A β accumulation and hydrolysis. Su et al. (72) used mononuclear RNA sequencing to generate transcriptome maps of the human hippocampus throughout the postnatal life cycle, and further characterized the spatiotemporal heterogeneity of GFAP rich astrocyte subsets in hippocampal formation using immunohistochemistry, which significantly expanded the understanding of human glial cell diversity, population dy namics and AD disorders in the postnatal life cycle, and provided a reference map for stem cell based glial cell differentiation. In view of the remarkable heterogeneity of astrocytes in the cortex and other regions, future studies exploring the characterization of astrocytes in the white matter and their relationship to neurodegeneration will be of even greater interest (73).

Microglia are resident macrophages in the central nervous system (CNS). It has the characteristics of multiple synapses and plasticity. It is an innate immune effector cell in the central nervous system and plays an extremely important role in the physiological process of the central nervous system. Tay et al. (74) identified some microglia subsets in the mouse brain, one of which is a unique transient microglia subset found at the beginning of disease recovery, characterized by the *FNX* dependent upregulation of *APOE* corresponding to no change in Trem2 expression. The researchers also elaborated that C9 microglia, which upregulates *APOE*, supports the opening of TREM2-APOE pathway in the recovery process to drive the unstable microglia phenotype, which may provide a new target for regulating microglia mediated brain health recovery. Li et al. (37) used deep single cells RNA sequencing (scRNA seq), found that most adult microglia expressing similar genes in mouse brain transcriptome stably. In contrast, microglia in early postnatal mice are more heterogeneous. In the early postnatal microglia, researchers found a proliferation region associated microglia (PAM) subpopulation, which mainly exists in the developing white matter and has amoeba morphology and metabolic activity. It shares the same genetic characteristics with degenerative disease associated microglia (DAM). Leaving the study on mice, Gupta and Kuznicki (75) used scRNA seq to explore the heterogeneity of microglia from healthy and neuropathological human tissues. This study found 13 different, time - and region dependent microglia clusters in the brains of healthy people and patients. Ten of these 13 clusters exist during development, two exist during demyelination and myelin regeneration, and one exists during neurodegeneration. This can provide a deeper explanation for the demyelination process of neurodegenerative diseases. Alsema et al. (76) studied the transcriptome of microglia at the large cell and single cell levels in non dementia elderly people and AD donors using human cortical brain samples, and identified seven human microglia subsets with heterogeneous gene expression. Olah et al. (77) used single cell RNA sequencing to find some subpopulations rich in disease related genes and RNA characteristics. The existence of four subgroups of microglia was confirmed histologically. Through further research on microglia cluster 7, it was found that these clusters were rich in depleted genes in the cortex of Alzheimer’s disease patients. Cell heterogeneity between the two AD pathological processes was also found, cluster 2 and cluster 5 enriched genes related to beta amyloid, while PHF tau related genes were enriched in cluster 1, 2, 4, 7, 8, and 9. This indicates that different subsets of microglia may participate in different aspects of AD. In a study based on an AD mouse model, Hemonnot-Girard et al. (78) combined cell specific laser capture microdissection and RNA seq analysis to identify genes and gene networks that are dysfunctional in PAM or PCM at the three critical stages of the disease without preconceived concepts of molecular and/or functional changes, and found potential contributions of plaque related and microglial cells far away from the plaque. In addition, some researchers have combined the mouse model and postmortem human brain for research. Sousa et al. (51) used single-cell transcriptomics and multi-color flow cytometry to show a comprehensive overview of mouse microglia after LPS injection. They found that the steady-state characteristics of microglia were mainly lost in acute systemic inflammation, inflammation induced microglia separated into two different reactive states, and the characteristics of inflammation induced microglia were different from those related to neurodegenerative diseases. In the research combining mouse and human brain, Marschallinger et al. (79) found that aging microglia in hippocampus would accumulate grease drops. They called these cells lipid droplet accumulating microglia (LDAM) and found that they were preferentially located around amyloid plaques in AD brain transcriptome analysis. It shows that LDAM may have proinflammatory effect, and there are also defects in phagocytosis. Silvin et al. (80) found that the disease associated microglia (DAM) population previously detected in the mouse Alzheimer’s disease model actually includes two different cell lineages: the embryonic derived trigger receptor expressed on myeloid cell 2 (TREM2) dependent DAM expresses neuroprotective characteristics and the monocyte derived TREM2 expresses inflammatory macrophages (DIM) of disease. These two different populations seem to be conservative in the human brain. Keren Shaul et al. (81) introduced the results of the scRNA seq experiment in this article, in which the microglia of wild-type mice, 5XFAD mice (AD model) and AD patients were evaluated. This study revealed a specific microglial subtype associated with neurodegeneration, characterized by increased expression of Cst7 and Lpl, indicating a specific increase in phagocytosis and lipid metabolism. The results of this study also showed that there was a relationship between the up-regulated expression of these genes and the two-step process of Trem2 activation in these microglia. In addition, these microglia subtypes have been found to be associated with amyotrophic lateral sclerosis (ALS), and they may be associated with more neurodegenerative diseases.

Oligodendrocytes wrap around axons in the central nervous system, form an insulated myelin sheath structure, assist in the jumping and efficient transmission of bioelectrical signals, and maintain and protect the normal function of neurons. Another part of researchers focus their attention here. Because APOE4 is a strong genetic risk of AD, Blanchard et al. (82) used scRNA-seq to study the relationship between human APOE4, myelination and memory deficit, and found that APOE4 impairs myelination through cholesterol imbalance in oligodendrocytes. This suggests that interventions to improve and promote cholesterol transport may enhance myelination of oligodendrocytes and alleviate cognitive deficits associated with APOE4 and AD. Lee et al. (83) sequenced 13 cell types in three different mouse models of Alzheimer’s disease (AD) to capture the pathological effects of tau only, amyloid only or combined tau amyloid, from which they determined two different transcriptional states of oligodendrocytes in different disease models and their spatial distribution.

Neurons are the basic units of the structure, function and genesis of the nervous system, and many researchers focus on such cells. Mathys et al. (84) sequenced a large number of human brain tissues with and without AD, from which 76 cell types were identified, including astrocytes, region-specific subtypes of excitatory neurons, and thalamus-specific populations of atypical inhibitory interneurons. Vulnerable populations of excitatory and inhibitory neurons in specific brain regions of AD patients were also identified. Li et al. (37) used Ion AmpliSeq to obtain gene expression data from a single neuron or a collection of neurons in the human brain. They found that the number of CA1 neurons in AD hippocampus with genes related to the olfactory system continued to decrease, especially in the olfactory receptor family. In addition to olfactory receptors, they also provided evidence that receptors (but not transmitters) of other neurotransmitters (dopamine, GABA and 5-hydroxytriamine receptors) are also down regulated in AD hippocampal neurons. These results may have therapeutic significance: improving the sensitivity of receptors may be more effective in clinical practice than simply increasing the level of neurotransmitters themselves. Deng et al. (85) found 9 and 11 excitatory neuron subsets in the human entorhinal cortex and superior frontal gyrus, respectively, and described region specific genes. The authors found neuronal subsets in these two regions, which are more likely to degenerate in the early and late stages, indicating their susceptibility to disease. This can be a basis for diagnosis and treatment. In addition, each cell in the nervous system is not an isolated individual, and the pathological changes of the nervous system will be reflected in a variety of cells. Sun et al. (86) identified three phenotypes of astrocytes and microglia, expanding from the past classification of “homeostasis and reactivity” to three classifications including “intermediate type.” Using snRNA seq, researchers found that reactive microglia not only expressed all reactive markers at a high level, but also expressed high levels of steady-state markers. The intermediate type expresses medium or low reactivity and steady-state markers, and applies the research results to high signal strength data (gradient enhancer) or machine learning models directly applied to image features (convolutional neural network), which is helpful to accurately distinguish control and AD diagnosis at the single cell level. The entorhinal cortex is closely related to human cognitive function, and it is also one of the first cortical regions to describe the neurofibrillary inclusion bodies and neuron loss in early AD. Grubman et al. (87) demonstrated the heterogeneity behind this early affected region by using postmortem human brain. It reveals how transcriptional changes of specific cell subsets are related to Alzheimer’s disease. For example, in AD patients, APOE is found to be down regulated in OPC and up regulated in astrocytes and microglia. In general, scRNA seq has played an important role in the molecular subtype analysis of diseases and in identifying new mechanisms and therapeutic targets. At present, three major molecular subtypes of AD have been identified through monocytomics. Each subtype is related to tau mediated neurodegeneration, amyloid beta neuroinflammation, synaptic signal transduction, immune activity, mitochondrial tissue and myelin sheath formation (88). On the other hand, the application of single cell multiomics also requires the ability to process large quantities of multiomics data. In view of this, researchers have conducted many meaningful multiomics data integration and analysis in the past research, and some meaningful markers and computational models have also been found and established in this process. Lee et al. (89) conducted an integrated analysis using multi group data generated from substantia nigra (SN). SN is the brain region most affected by PD. Mononuclear RNA sequencing (snRNA seq) and chromatin accessibility (snaTAC seq) established the cell type distinguishing transcriptome and epigenome of PD and control SN. It also revealed that the specific imbalance of dopaminergic neurons and glial cells (including oligodendrocytes and microglia) in cRE has a strong transcriptional effect on PD related genes. In addition, Elkjaer et al. (90) studied multiple sclerosis by combining transcriptome and proteome, explaining the mechanism and role of oligodendrocytes, microglia, astrocytes and neurons in the pathogenesis and development of disease, and also indicating that chronic active disease is the most unique type of disease in multiple sclerosis. In terms of computing model, Jin et al. (91) developed a computing pipeline scGRNom, which is used to integrate multi omics data and predict gene regulatory networks (GRNs). These networks link TFs, non-coding regulatory elements (such as enhancers) and target genes. By applying to human brain single cell multiomics data (such as epigenomics and single cell transcriptomics), researchers predicted the cellular GRN of neurons (such as excitability and inhibition) and glial cell types (such as microglia and oligodendrocytes). In addition, the research of Li et al. (92) introduced STREAM, which is a new method using Steiner forest problem model, mixed bicluster pipeline and submodulation optimization. It can jointly analyze single cell transcriptome (snRNA seq) and chromatin accessibility (snATAC seq) data, and infer enhancer driven gene regulatory network (eGRN) from them. Compared with existing methods, STREAM shows enhanced performance in TF recovery, TF enhancer connection prediction and enhancer gene relationship discovery.

## 5 Conclusion

Single-cell omics offers a novel approach to the study of neurodegenerative diseases, allowing researchers to identify the targets and mechanisms of disease occurrence from the perspective of cellular heterogeneity. This approach also facilitates the development of new disease models and the proposal of new diagnostic and therapeutic methods.

### 5.1 Limitations

This study used bibliometric visualization to analyze the research on single-cell omics in neurodegenerative diseases over the past 10 years. However, this study inevitably has certain limitations. Firstly, the data analyzed in this study only comes from the WOSCC database and does not include data from other databases such as PubMed, Cochrane Library, and Google Scholar. Different databases can affect bibliometric results. It has been shown that utilizing combined databases may produce different results compared to studies based on individual citation databases (93), which may make our article more comprehensive. Whereas it has been shown that national output and impact obtained from WOS and PubMed are highly correlated, but PubMed has a narrower field of research (94). The most important point is that the PubMed database cannot export citation-related data. And citation-related data can help us to understand the trend of the research object well. Also, both Scopus and WOS have research field and language preferences, but Scopus is currently limited to recent articles (published after 1995) (95). Therefore, although WOSCC has its comprehensiveness and reliability, there are more or less omissions in its database. Secondly, we excluded literature outside the English language, which may introduce biases. We searched 596 documents and excluded 1 non-English language document (German) from the search process. We searched in Mesh and found 117 articles, with English language literature making up the vast majority (117 articles). Due to the largest number of articles in English, we have chosen only English articles for statistical purposes. Choosing to count all languages may be more comprehensive. Finally, the data in this study may vary in various aspects, such as the same research institution using different names at different time stages. At the same time, some of the good quality articles may have received a low citation rate because of the recentness of their publication. These are issues that should be considered in a bibliometric analysis.

## Data Availability

The original contributions presented in the study are included in the article/Supplementary material, further inquiries can be directed to the corresponding authors.

## References

[1] Kritsilis M Rizou SV Koutsoudaki PN Evangelou K Gorgoulis VG Papadopoulos D. Cellular senescence and neurodegenerative disease. Int J Mol Sci. (2018) 19:2937. doi: 10.3390/ijms19102937, PMID: 30261683 PMC6213570

[2] Li X Feng X Sun X Hou N Han F Liu Y. Global, regional, and national burden of Alzheimer’s disease and other dementias, 1990–2019. Front Aging Neurosci. (2022) 14:937486. doi: 10.3389/fnagi.2022.937486, PMID: 36299608 PMC9588915

[3] Forsell C Björk BF Lilius L Axelman K Fabre SF Fratiglioni L . Genetic association to the amyloid plaque associated protein gene COL25A1 in Alzheimer’s disease. Neurobiol Aging. (2010) 31:409–15. doi: 10.1016/j.neurobiolaging.2008.04.009 18501477

[4] Dugger BN Dickson DW. Pathology of neurodegenerative diseases. Cold Spring Harb Perspect Biol. (2017) 9:a028035. doi: 10.1101/cshperspect.a028035, PMID: 28062563 PMC5495060

[5] Gale SA Acar D Daffner KR. Dementia. Am J Med. (2018) 131:1161–9. doi: 10.1016/j.amjmed.2018.01.022 29425707

[6] Haass C Selkoe DJ. Soluble protein oligomers in neurodegeneration: lessons from the Alzheimer’s amyloid beta-peptide. Nat Rev Mol Cell Biol. (2007) 8:101–12. doi: 10.1038/nrm2101, PMID: 17245412

[7] Cuevas-Diaz Duran R Carlos Gonzalez-Orozco J Velasco I Wu JQ. Single-cell and single-nuclei RNA sequencing as powerful tools to decipher cellular heterogeneity and dysregulation in neurodegenerative diseases. Front Cell Dev Biol. (2022) 10:884748. doi: 10.3389/fcell.2022.884748, PMID: 36353512 PMC9637968

[8] Marogianni C Sokratous M Dardiotis E Hadjigeorgiou GM Bogdanos D Xiromerisiou G. Neurodegeneration and inflammation-an interesting interplay in Parkinson’s disease. Int J Mol Sci. (2020) 21:8421. doi: 10.3390/ijms21228421, PMID: 33182554 PMC7697354

[9] Lu T Aron L Zullo J Pan Y Kim H Chen Y . REST and stress resistance in ageing and Alzheimer’s disease. Nature. (2014) 507:448–54. doi: 10.1038/nature13163, PMID: 24670762 PMC4110979

[10] Kampmann M. Molecular and cellular mechanisms of selective vulnerability in neurodegenerative diseases. Nat Rev Neurosci. (2024) 25:351–71. doi: 10.1038/s41583-024-00806-0, PMID: 38575768

[11] Davies P Maloney AJ. Selective loss of central cholinergic neurons in Alzheimer’s disease. Lancet. (1976) 2:1403. doi: 10.1016/s0140-6736(76)91936-x, PMID: 63862

[12] Gao C Jiang J Tan Y Chen S. Microglia in neurodegenerative diseases: mechanism and potential therapeutic targets. Signal Transduct Target Ther. (2023) 8:359. doi: 10.1038/s41392-023-01588-0 37735487 PMC10514343

[13] Huo L Jiao Li J Chen L Yu Z Hutvagner G Li J. Single-cell multiomics sequencing: application trends, COVID-19, data analysis issues and prospects. Brief Bioinform. (2021) 22:bbab229. doi: 10.1093/bib/bbab229, PMID: 34111889 PMC8344433

[14] Kolodziejczyk AA Kim JK Svensson V Marioni JC Teichmann SA. The technology and biology of single-cell RNA sequencing. Mol Cell. (2015) 58:610–20. doi: 10.1016/j.molcel.2015.04.005 26000846

[15] Verheijen BM Vermulst M van Leeuwen FW. Somatic mutations in neurons during aging and neurodegeneration. Acta Neuropathol. (2018) 135:811–26. doi: 10.1007/s00401-018-1850-y, PMID: 29705908 PMC5954077

[16] Tejwani L Ravindra NG Lee C Cheng Y Nguyen B Luttik K . Longitudinal single-cell transcriptional dynamics throughout neurodegeneration in SCA1. Neuron. (2024) 112:362–383.e15. doi: 10.1016/j.neuron.2023.10.039 38016472 PMC10922326

[17] Awuah WA Ahluwalia A Ghosh S Roy S Tan JK Adebusoye FT . The molecular landscape of neurological disorders: insights from single-cell RNA sequencing in neurology and neurosurgery. Eur J Med Res. (2023) 28:529. doi: 10.1186/s40001-023-01504-w, PMID: 37974227 PMC10652629

[18] Baysoy A Bai Z Satija R Fan R. The technological landscape and applications of single-cell multiomics. Nat Rev Mol Cell Biol. (2023) 24:695–713. doi: 10.1038/s41580-023-00615-w, PMID: 37280296 PMC10242609

[19] Mathys H Davila-Velderrain J Peng Z Gao F Mohammadi S Young JZ . Single-cell transcriptomic analysis of Alzheimer’s disease. Nature. (2019) 570:332–7. doi: 10.1038/s41586-019-1195-2, PMID: 31042697 PMC6865822

[20] Ninkov A Frank JR Maggio LA. Bibliometrics: methods for studying academic publishing. Perspect Med Educ. (2022) 11:173–6. doi: 10.1007/s40037-021-00695-4, PMID: 34914027 PMC9240160

[21] Kokol P Blažun Vošner H Završnik J. Application of bibliometrics in medicine: a historical bibliometrics analysis. Health Inf Libr J. (2021) 38:125–38. doi: 10.1111/hir.12295 31995273

[22] Chen L Wan Y Yang T Zhang Q Zeng Y Zheng S . Bibliometric and visual analysis of single-cell sequencing from 2010 to 2022. Front Genet. (2023) 14:1285599. doi: 10.3389/fgene.2023.1285599 38274109 PMC10808606

[23] Xiao H Tang J Zhang F Liu L Zhou J Chen M . Global trends and performances in diabetic retinopathy studies: a bibliometric analysis. Front Public Health. (2023) 11:1128008. doi: 10.3389/fpubh.2023.1128008, PMID: 37124794 PMC10136779

[24] Duan Y Zhang P Zhang T Zhou L Yin R. Characterization of global research trends and prospects on platinum-resistant ovarian cancer: a bibliometric analysis. Front Oncol. (2023) 13:1151871. doi: 10.3389/fonc.2023.1151871, PMID: 37342181 PMC10277726

[25] Zhang L Yao Q Hu J Qiu B Xiao Y Zhang Q . Hotspots and trends of microglia in Alzheimer’s disease: a bibliometric analysis during 2000–2022. Eur J Med Res. (2024) 29:75. doi: 10.1186/s40001-023-01602-9, PMID: 38268044 PMC10807212

[26] Zhong D Li Y Huang Y Hong X Li J Jin R. Molecular mechanisms of exercise on cancer: a bibliometrics study and visualization analysis via CiteSpace. Front Mol Biosci. (2021) 8:797902. doi: 10.3389/fmolb.2021.797902, PMID: 35096970 PMC8794585

[27] van Eck NJ Waltman L. Software survey: VOSviewer, a computer program for bibliometric mapping. Scientometrics. (2010) 84:523–38. doi: 10.1007/s11192-009-0146-3, PMID: 20585380 PMC2883932

[28] Liao Z Wei W Yang M Kuang X Shi J. Academic publication of neurodegenerative diseases from a bibliographic perspective: a comparative scientometric analysis. Front Aging Neurosci. (2021) 13:722944. doi: 10.3389/fnagi.2021.722944, PMID: 34803653 PMC8601281

[29] Chen CM Leydesdorff L. Patterns of connections and movements in dual-map overlays: a new method of publication portfolio analysis. J Assoc Inf Sci Technol. (2014) 65:334–51. doi: 10.1002/asi.22968

[30] Zhang J Song LX Xu LY Fan YX Wang T Tian WD . Knowledge domain and emerging trends in ferroptosis research: a bibliometric and knowledge-map analysis. Front Oncol. (2021) 11:686726. doi: 10.3389/fonc.2021.686726, PMID: 34150654 PMC8209495

[31] Selkoe DJ. Alzheimer’s disease: genes, proteins, and therapy. Physiol Rev. (2001) 81:741–66. doi: 10.1152/physrev.2001.81.2.741 11274343

[32] Marx V. Method of the year: spatially resolved transcriptomics. Nat Methods. (2021) 18:9–14. doi: 10.1038/s41592-020-01033-y 33408395

[33] Jovic D Liang X Zeng H Lin L Xu F Luo Y. Single-cell RNA sequencing technologies and applications: a brief overview. Clin Transl Med. (2022) 12:e694. doi: 10.1002/ctm2.694, PMID: 35352511 PMC8964935

[34] Piwecka M Rajewsky N Rybak-Wolf A. Single-cell and spatial transcriptomics: deciphering brain complexity in health and disease. Nat Rev Neurol. (2023) 19:346–62. doi: 10.1038/s41582-023-00809-y, PMID: 37198436 PMC10191412

[35] Masarapu Y Cekanaviciute E Andrusivova Z Westholm JO Bjorklund A Fallegger R . Spatially resolved multiomics on the neuronal effects induced by spaceflight in mice. Nat Commun. (2024) 15:4778. doi: 10.1038/s41467-024-48916-8, PMID: 38862479 PMC11166911

[36] Hammond TR Dufort C Dissing-Olesen L Giera S Young A Wysoker A . Single-cell RNA sequencing of microglia throughout the mouse lifespan and in the injured brain reveals complex cell-state changes. Immunity. (2019) 50:253–271.e6. doi: 10.1016/j.immuni.2018.11.004, PMID: 30471926 PMC6655561

[37] Li QY Cheng ZL Zhou L Darmanis S Neff NF Okamoto J . Developmental heterogeneity of microglia and brain myeloid cells revealed by deep single-cell RNA sequencing. Neuron. (2019) 101:207–223.e10. doi: 10.1016/j.neuron.2018.12.006, PMID: 30606613 PMC6336504

[38] Shadrina MI Slominsky PA. Genetic architecture of Parkinson’s disease. Biochemistry. (2023) 88:417–33. doi: 10.1134/s0006297923030100 37076287

[39] Stuart T Butler A Hoffman P Hafemeister C Papalexi E Mauck WM . Comprehensive integration of single-cell data. Cell. (2019) 177:1888–1902.e21. doi: 10.1016/j.cell.2019.05.031, PMID: 31178118 PMC6687398

[40] Wang Y Jiang R Li M Wang Z Yang Y Sun L. Characteristics of T cells in single-cell datasets of peripheral blood and cerebrospinal fluid in Alzheimer’s disease patients. J Alzheimers Dis. (2024) 99:S265–80. doi: 10.3233/JAD-230784 38043012 PMC11091562

[41] Cheng C Chen W Jin H Chen X. A review of single-cell RNA-seq annotation, integration, and cell–cell communication. Cells. (2023) 12:1970. doi: 10.3390/cells12151970, PMID: 37566049 PMC10417635

[42] Chen X Firulyova M Manis M Herz J Smirnov I Aladyeva E . Microglia-mediated T cell infiltration drives neurodegeneration in tauopathy. Nature. (2023) 615:668–77. doi: 10.1038/s41586-023-05788-0, PMID: 36890231 PMC10258627

[43] Weng W Fu J Cheng F Wang Y Zhang J. Integrated bulk and single-cell RNA-sequencing reveals the effects of circadian rhythm disruption on the metabolic reprogramming of CD4^+^ T cells in Alzheimer’s disease. Mol Neurobiol. (2024) 61:6013–30. doi: 10.1007/s12035-023-03907-6, PMID: 38265551

[44] Gate D Saligrama N Leventhal O Yang AC Unger MS Middeldorp J . Clonally expanded CD8 T cells patrol the cerebrospinal fluid in Alzheimer’s disease. Nature. (2020) 577:399–404. doi: 10.1038/s41586-019-1895-7 31915375 PMC7445078

[45] Schafflick D Xu CA Hartlehnert M Cole M Schulte-Mecklenbeck A Lautwein T . Integrated single cell analysis of blood and cerebrospinal fluid leukocytes in multiple sclerosis. Nat Commun. (2020) 11:247. doi: 10.1038/s41467-019-14118-w, PMID: 31937773 PMC6959356

[46] Jorfi M Park J Hall CK Jerry Lin C-C Chen M von Maydell D . Infiltrating CD8^+^ T cells exacerbate Alzheimer’s disease pathology in a 3D human neuroimmune axis model. Nat Neurosci. (2023) 26:1489–504. doi: 10.1038/s41593-023-01415-3, PMID: 37620442 PMC11184920

[47] Su W Saravia J Risch I Rankin S Guy C Chapman NM . CXCR6 orchestrates brain CD8^+^ T cell residency and limits mouse Alzheimer’s disease pathology. Nat Immunol. (2023) 24:1735–47. doi: 10.1038/s41590-023-01604-z, PMID: 37679549 PMC11102766

[48] Beltrán E Gerdes LA Hansen J Flierl-Hecht A Krebs S Blum H . Early adaptive immune activation detected in monozygotic twins with prodromal multiple sclerosis. J Clin Invest. (2019) 129:4758–68. doi: 10.1172/JCI128475 31566584 PMC6819125

[49] Piehl N van Olst L Ramakrishnan A Teregulova V Simonton B Zhang Z . Cerebrospinal fluid immune dysregulation during healthy brain aging and cognitive impairment. Cell. (2022) 185:5028–5039.e13. doi: 10.1016/j.cell.2022.11.019, PMID: 36516855 PMC9815831

[50] Rosen SF Soung AL Yang W Ai S Kanmogne M Davé VA . Single-cell RNA transcriptome analysis of CNS immune cells reveals CXCL16/CXCR6 as maintenance factors for tissue-resident T cells that drive synapse elimination. Genome Med. (2022) 14:108. doi: 10.1186/s13073-022-01111-0, PMID: 36153630 PMC9509564

[51] Sousa C Golebiewska A Poovathingal SK Kaoma T Pires-Afonso Y Martina S . Single-cell transcriptomics reveals distinct inflammation-induced microglia signatures. EMBO Rep. (2018) 19:e46171. doi: 10.15252/embr.201846171, PMID: 30206190 PMC6216255

[52] Maeda C Tsuruta F. Molecular basis of neuronal and microglial states in the aging brain and impact on cerebral blood vessels. Int J Mol Sci. (2024) 25:4443. doi: 10.3390/ijms25084443, PMID: 38674028 PMC11049950

[53] Hou Y Dan X Babbar M Wei Y Hasselbalch SG Croteau DL . Ageing as a risk factor for neurodegenerative disease. Nat Rev Neurol. (2019) 15:565–81. doi: 10.1038/s41582-019-0244-7, PMID: 31501588

[54] Shi Y Zhu R. Analysis of damage-associated molecular patterns in amyotrophic lateral sclerosis based on ScRNA-seq and bulk RNA-seq data. Front Neurosci. (2023) 17:1259742. doi: 10.3389/fnins.2023.1259742, PMID: 37942135 PMC10628000

[55] Jian C Wei L Mo R Li R Liang L Chen L . Microglia mediate the occurrence and development of Alzheimer’s disease through ligand-receptor axis communication. Front Aging Neurosci. (2021) 13:731180. doi: 10.3389/fnagi.2021.731180, PMID: 34616287 PMC8488208

[56] Mifflin L Hu Z Dufort C Hession CC Walker AJ Niu K . A RIPK1-regulated inflammatory microglial state in amyotrophic lateral sclerosis. Proc Natl Acad Sci USA. (2021) 118:e2025102118. doi: 10.1073/pnas.2025102118, PMID: 33766915 PMC8020785

[57] Lee CY Riffle D Xiong Y Momtaz N Lei Y Pariser JM . Characterizing dysregulations via cell-cell communications in Alzheimer’s brains using single-cell transcriptomes. BMC Neurosci. (2024) 25:24. doi: 10.1186/s12868-024-00867-y, PMID: 38741048 PMC11089696

[58] Garden GA La Spada AR. Intercellular (Mis)communication in neurodegenerative disease. Neuron. (2012) 73:886–901. doi: 10.1016/j.neuron.2012.02.017, PMID: 22405200 PMC3334539

[59] Li J Jaiswal MK Chien J-F Kozlenkov A Jung J Zhou P . Divergent single cell transcriptome and epigenome alterations in ALS and FTD patients with C9orf72 mutation. Nat Commun. (2023) 14:5714. doi: 10.1038/s41467-023-41033-y 37714849 PMC10504300

[60] Sun Y Zhang H Zhang X Wang W Chen Y Cai Z . Promotion of astrocyte-neuron glutamate-glutamine shuttle by SCFA contributes to the alleviation of Alzheimer’s disease. Redox Biol. (2023) 62:102690. doi: 10.1016/j.redox.2023.102690, PMID: 37018970 PMC10122027

[61] Zhang C Tan G Zhang Y Zhong X Zhao Z Peng Y . Comprehensive analyses of brain cell communications based on multiple scRNA-seq and snRNA-seq datasets for revealing novel mechanism in neurodegenerative diseases. CNS Neurosci Ther. (2023) 29:2775–86. doi: 10.1111/cns.14280, PMID: 37269061 PMC10493674

[62] Szebényi K Wenger LMD Sun Y Dunn AWE Limegrover CA Gibbons GM . Human ALS/FTD brain organoid slice cultures display distinct early astrocyte and targetable neuronal pathology. Nat Neurosci. (2021) 24:1542–54. doi: 10.1038/s41593-021-00923-4 34675437 PMC8553627

[63] Siddiqui T Cosacak MI Popova S Bhattarai P Yilmaz E Lee AJ . Nerve growth factor receptor (Ngfr) induces neurogenic plasticity by suppressing reactive astroglial Lcn2/Slc22a17 signaling in Alzheimer’s disease. npj Regen Med. (2023) 8:33. doi: 10.1038/s41536-023-00311-5, PMID: 37429840 PMC10333226

[64] Wheeler MA Clark IC Tjon EC Li Z Zandee SEJ Couturier CP . MAFG-driven astrocytes promote CNS inflammation. Nature. (2020) 578:593–9. doi: 10.1038/s41586-020-1999-0, PMID: 32051591 PMC8049843

[65] Cano A Ettcheto M Bernuz M Puerta R De AEE Sánchez-López E . Extracellular vesicles, the emerging mirrors of brain physiopathology. Int J Biol Sci. (2023) 19:721–43. doi: 10.7150/ijbs.79063, PMID: 36778117 PMC9910004

[66] Jang J Yeo S Baek S Jung HJ Lee MS Choi SH . Abnormal accumulation of extracellular vesicles in hippocampal dystrophic axons and regulation by the primary cilia in Alzheimer’s disease. Acta Neuropathol Commun. (2023) 11:142. doi: 10.1186/s40478-023-01637-3, PMID: 37667395 PMC10478284

[67] Cosacak MI Bhattarai R Reinhardt S Petzold A Dahl A Zhang Y . Single-cell transcriptomics analyses of neural stem cell heterogeneity and contextual plasticity in a zebrafish brain model of amyloid toxicity. Cell Rep. (2019) 27:1307–1318.e3. doi: 10.1016/j.celrep.2019.03.090 31018142

[68] Wang S Karikomi M AL ML Nie Q eds. Cell lineage and communication network inference via optimization for single-cell transcriptomics. Nucleic Acids Res. (2024) 47:e66. doi: 10.1093/nar/gkz204 PMC6582411 30923815

[69] Pozojevic J Spielmann M. Single-cell sequencing in neurodegenerative disorders. Mol Diagn Ther. (2023) 27:553–61. doi: 10.1007/s40291-023-00668-9, PMID: 37552451 PMC10435411

[70] Smith AM Davey K Tsartsalis S Khozoie C Fancy N Tang SS . Diverse human astrocyte and microglial transcriptional responses to Alzheimer’s pathology. Acta Neuropathol. (2022) 143:75–91. doi: 10.1007/s00401-021-02372-6, PMID: 34767070 PMC8732962

[71] Habib N McCabe C Medina S Varshavsky M Kitsberg D Dvir-Szternfeld R . Disease-associated astrocytes in Alzheimer’s disease and aging. Nat Neurosci. (2020) 23:701–6. doi: 10.1038/s41593-020-0624-8, PMID: 32341542 PMC9262034

[72] Su YJ Zhou Y Bennett ML Li SY Carceles-Cordon M Lu L . A single-cell transcriptome atlas of glial diversity in the human hippocampus across the postnatal lifespan. Cell Stem Cell. (2022) 29:1594–1610.e8. doi: 10.1016/j.stem.2022.09.010, PMID: 36332572 PMC9844262

[73] Köhler S Winkler U Hirrlinger J. Heterogeneity of astrocytes in grey and white matter. Neurochem Res. (2021) 46:3–14. doi: 10.1007/s11064-019-02926-x 31797158

[74] Tay TL Sagar Dautzenberg J Sagar DJ Grün D Prinz M. Unique microglia recovery population revealed by single-cell RNAseq following neurodegeneration. Acta Neuropathol Commun. (2018) 6:87. doi: 10.1186/s40478-018-0584-3 30185219 PMC6123921

[75] Gupta RK Kuznicki J. Biological and medical importance of cellular heterogeneity deciphered by single-cell RNA sequencing. Cells. (2020) 9:1751. doi: 10.3390/cells9081751, PMID: 32707839 PMC7463515

[76] Alsema AM Jiang Q Kracht L Gerrits E Dubbelaar ML Miedema A . Profiling microglia from Alzheimer’s disease donors and non-demented elderly in acute human postmortem cortical tissue. Front Mol Neurosci. (2020) 13:134. doi: 10.3389/fnmol.2020.00134, PMID: 33192286 PMC7655794

[77] Olah M Menon V Habib N Taga MF Ma YY Yung CJ . Single cell RNA sequencing of human microglia uncovers a subset associated with Alzheimer’s disease. Nat Commun. (2020) 11:6129. doi: 10.1038/s41467-020-19737-2, PMID: 33257666 PMC7704703

[78] Hemonnot-Girard AL Meersseman C Pastore M Garcia V Linck N Rey C . Comparative analysis of transcriptome remodeling in plaque-associated and plaque-distant microglia during amyloid-β pathology progression in mice. J Neuroinflammation. (2022) 19:234. doi: 10.1186/s12974-022-02581-0, PMID: 36153535 PMC9508749

[79] Marschallinger J Iram T Zardeneta M Lee SE Lehallier B Haney MS . Lipid-droplet-accumulating microglia represent a dysfunctional and proinflammatory state in the aging brain. Nat Neurosci. (2020) 23:194–208. doi: 10.1038/s41593-019-0566-1 31959936 PMC7595134

[80] Silvin A Uderhardt S Piot C Da Mesquita S Yang K Geirsdottir L . Dual ontogeny of disease-associated microglia and disease inflammatory macrophages in aging and neurodegeneration. Immunity. (2022) 55:1448–1465.e6. doi: 10.1016/j.immuni.2022.07.004, PMID: 35931085

[81] Keren-Shaul H Spinrad A Weiner A Matcovitch-Natan O Dvir-Szternfeld R Ulland TK . A unique microglia type associated with restricting development of Alzheimer’s disease. Cell. (2017) 169:1276–1290.e17. doi: 10.1016/j.cell.2017.05.018, PMID: 28602351

[82] Blanchard JW Akay LA Davila-Velderrain J von Maydell D Mathys H Davidson SM . APOE4 impairs myelination via cholesterol dysregulation in oligodendrocytes. Nature. (2022) 611:769–79. doi: 10.1038/s41586-022-05439-w, PMID: 36385529 PMC9870060

[83] Lee SH Rezzonico MG Friedman BA Huntley MH Meilandt WJ Pandey S . TREM2-independent oligodendrocyte, astrocyte, and T cell responses to tau and amyloid pathology in mouse models of Alzheimer disease. Cell Rep. (2021) 37:110158. doi: 10.1016/j.celrep.2021.110158, PMID: 34965428

[84] Mathys H Boix CA Akay LA Xia Z Davila-Velderrain J Ng AP . Single-cell multiregion dissection of Alzheimer’s disease. Nature. (2024) 632:858–68. doi: 10.1038/s41586-024-07606-7, PMID: 39048816 PMC11338834

[85] Deng WJ Xing CH David R Mastroeni D Ning MM Lo EH . AmpliSeq transcriptome of laser captured neurons from Alzheimer brain: comparison of single cell versus neuron pools. Aging Dis. (2019) 10:1146–58. doi: 10.14336/ad.2019.0225, PMID: 31788328 PMC6844587

[86] Sun JJ Song YX Chen ZH Qiu JY Zhu SX Wu LC . Heterogeneity and molecular markers for CNS glial cells revealed by single-cell transcriptomics. Cell Mol Neurobiol. (2022) 42:2629–42. doi: 10.1007/s10571-021-01159-3, PMID: 34704168 PMC11421601

[87] Grubman A Chew G Ouyang JF Sun GZ Choo XY McLean C . A single-cell atlas of entorhinal cortex from individuals with Alzheimer’s disease reveals cell-type-specific gene expression regulation. Nat Neurosci. (2019) 22:2087–97. doi: 10.1038/s41593-019-0539-4, PMID: 31768052

[88] Neff RA Wang MH Vatansever S Guo L Ming C Wang Q . Molecular subtyping of Alzheimer’s disease using RNA sequencing data reveals novel mechanisms and targets. Sci Adv. (2021) 7:eabb5398. doi: 10.1126/sciadv.abb5398, PMID: 33523961 PMC7787497

[89] Lee AJ Kim C Park S Joo J Choi B Yang D . Characterization of altered molecular mechanisms in Parkinson’s disease through cell type-resolved multiomics analyses. Sci Adv. (2023) 9:eabo2467. doi: 10.1126/sciadv.abo2467, PMID: 37058563 PMC10104466

[90] Elkjaer ML Röttger R Baumbach J Illes Z. A systematic review of tissue and single cell transcriptome/proteome studies of the brain in multiple sclerosis. Front Immunol. (2022) 13:761225. doi: 10.3389/fimmu.2022.761225, PMID: 35309325 PMC8924618

[91] Jin T Rehani P Ying MF Huang JW Liu S Roussos P . scGRNom: a computational pipeline of integrative multiomics analyses for predicting cell-type disease genes and regulatory networks. Genome Med. (2021) 13:95. doi: 10.1186/s13073-021-00908-9, PMID: 34044854 PMC8161957

[92] Li Y Ma AJ Wang YZ Guo Q Wang CK Fu HJ . Enhancer-driven gene regulatory networks inference from single-cell RNA-seq and ATAC-seq data. Brief Bioinform. (2024) 25:bbae369. doi: 10.1093/bib/bbae369, PMID: 39082647 PMC11289686

[93] Nikolić D Ivanović D Ivanović L. An open-source tool for merging data from multiple citation databases. Scientometrics. (2024) 129:4573–95. doi: 10.1007/s11192-024-05076-2

[94] Archambault E Campbell D Gingras Y Lariviere V. Comparing of science bibliometric statistics obtained from the Web of Science and Scopus. J Am Soc Inf Sci Technol. (2009) 60:1320–6. doi: 10.1002/asi.21062

[95] Mongeon P Paul-Hus A. The journal coverage of Web of Science and Scopus: a comparative analysis. Scientometrics. (2016) 106:213–28. doi: 10.1007/s11192-015-1765-5

