# Supplementary figures and images for: Bibliometric and visual analysis of single-cell multiomics in neurodegenerative disease arrest studies
Source: Front Neurol. 2024 Oct 8;15:1450663. doi: 10.3389/fneur.2024.1450663 (PMC11493674; doi:10.3389/fneur.2024.1450663)

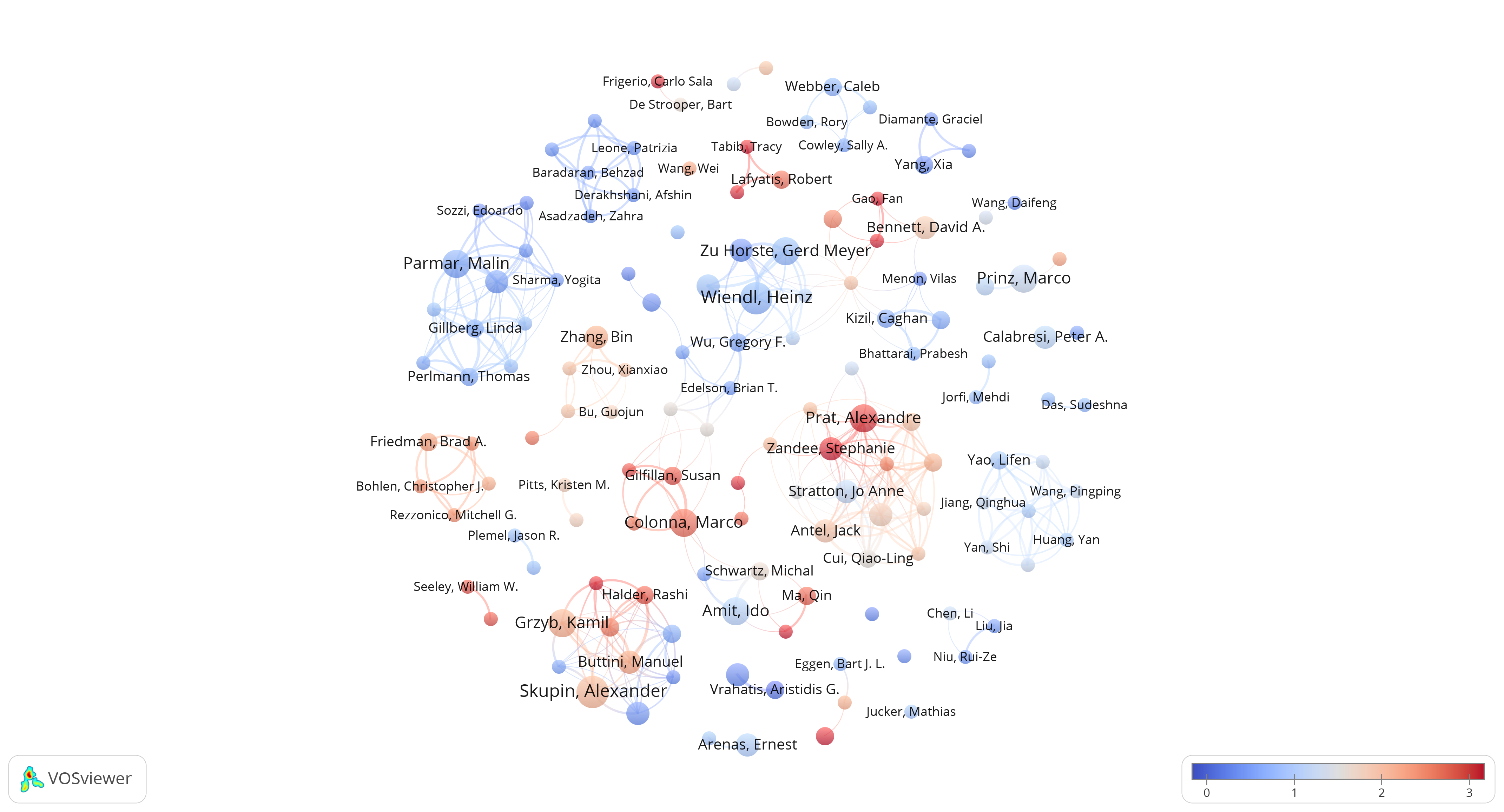

Supplement: Supplementary file 2 [file Image_1.PNG]

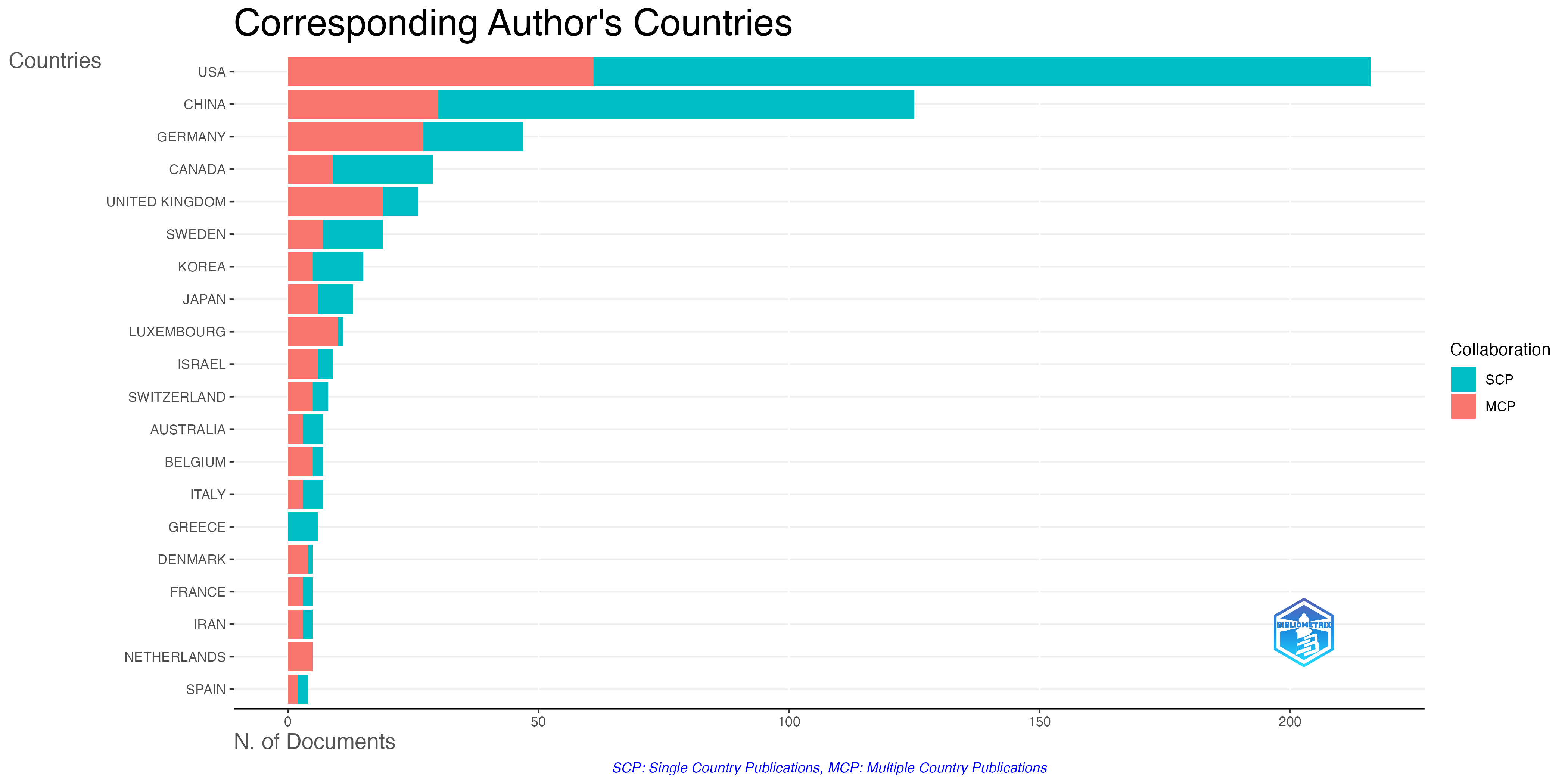

Supplement: Supplementary file 3 [file Image_2.PNG]

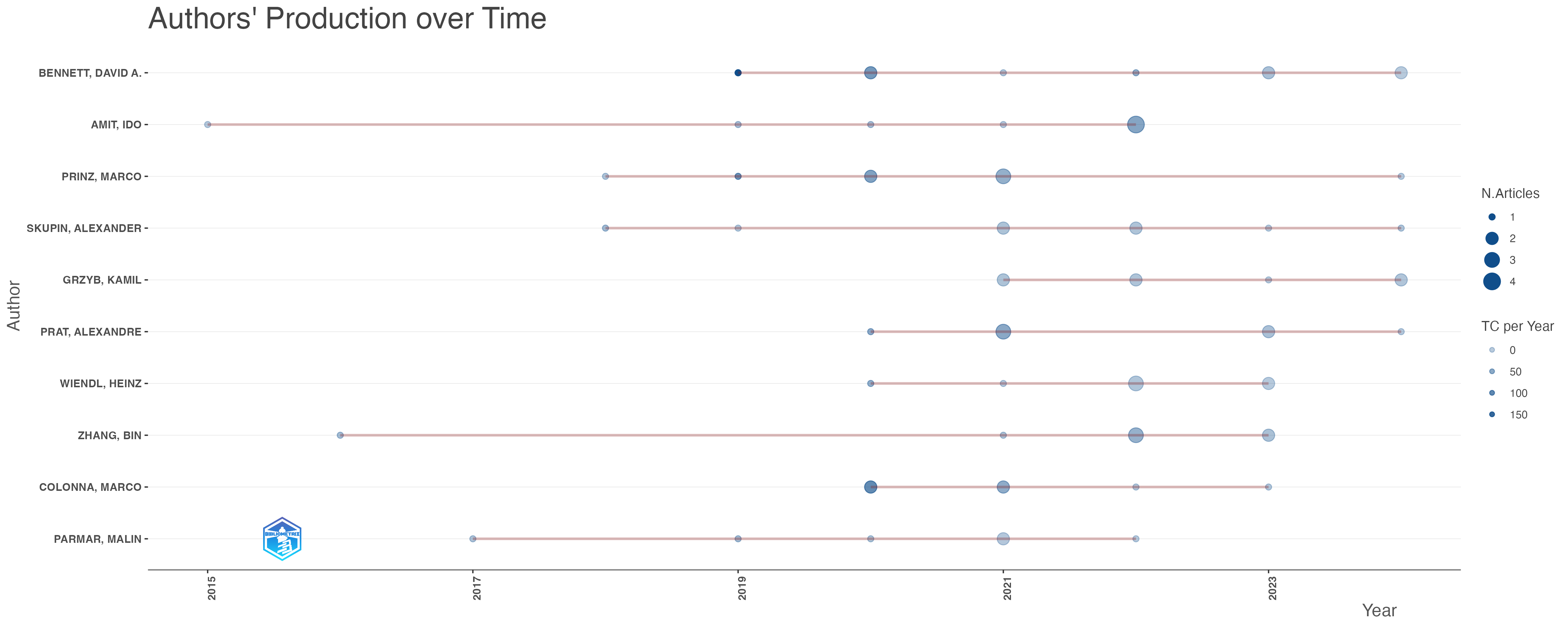

Supplement: Supplementary file 4 [file Image_3.PNG]

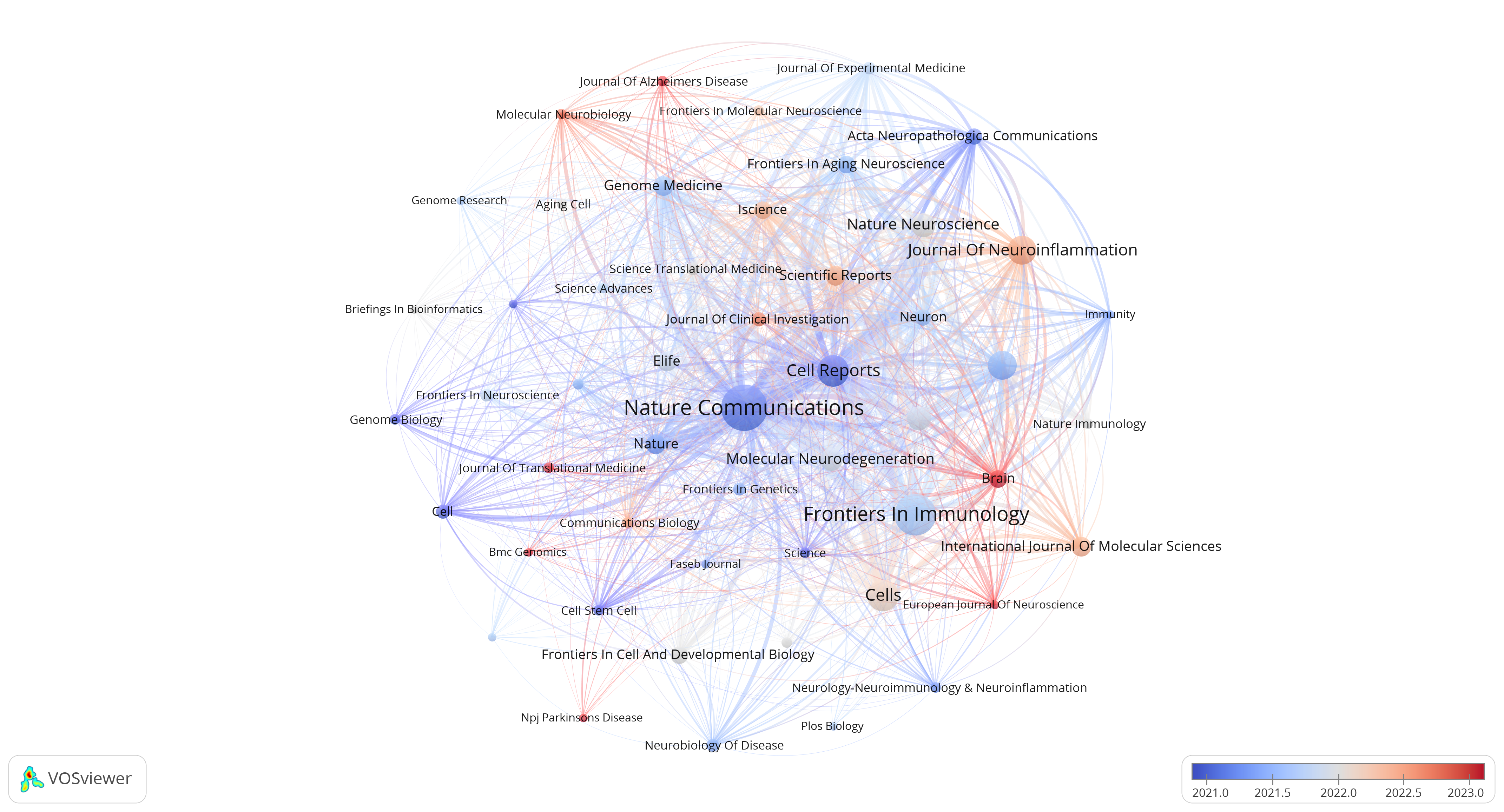

Supplement: Supplementary file 5 [file Image_4.PNG]

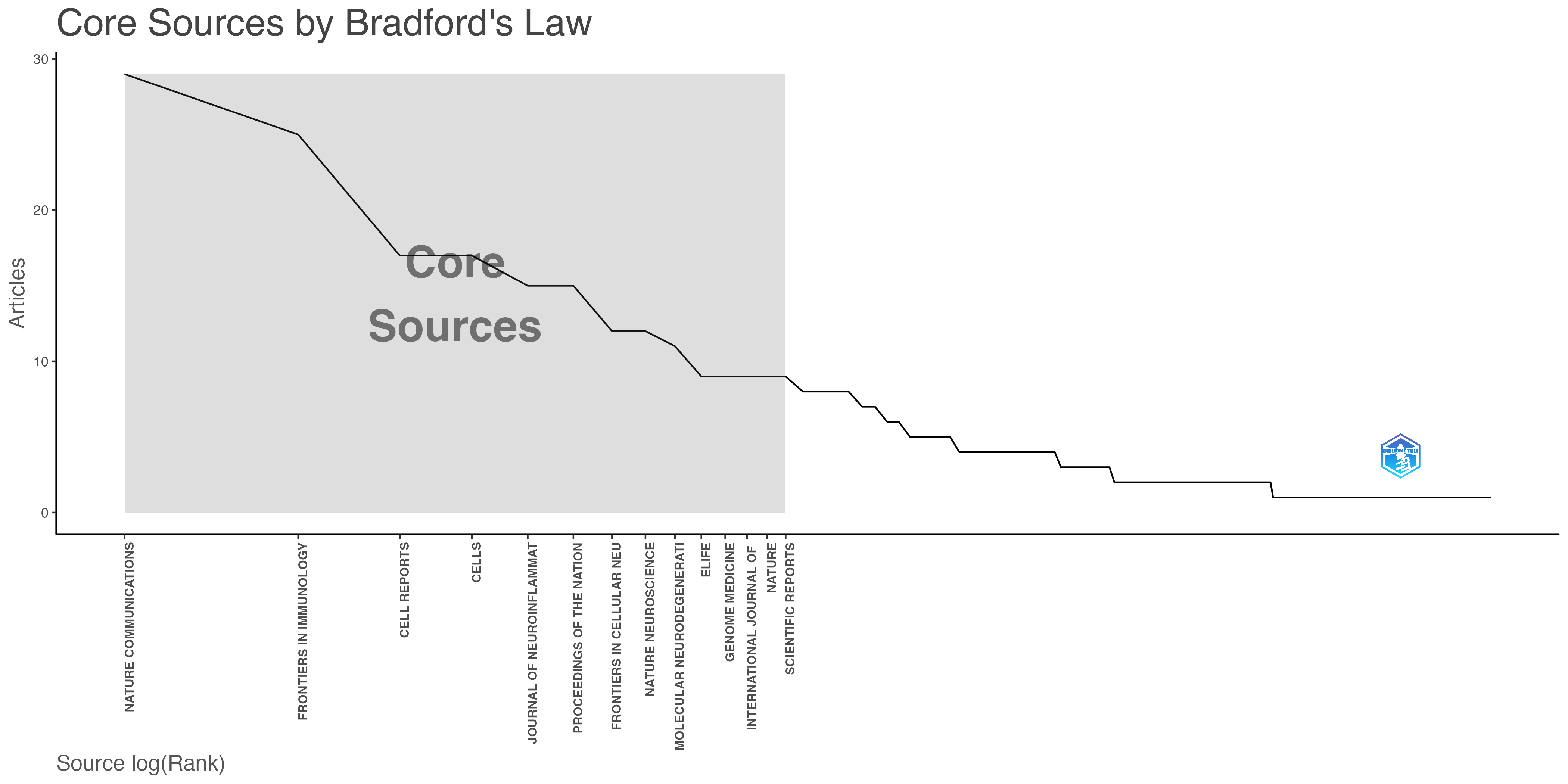

Supplement: Supplementary file 6 [file Image_5.PNG]

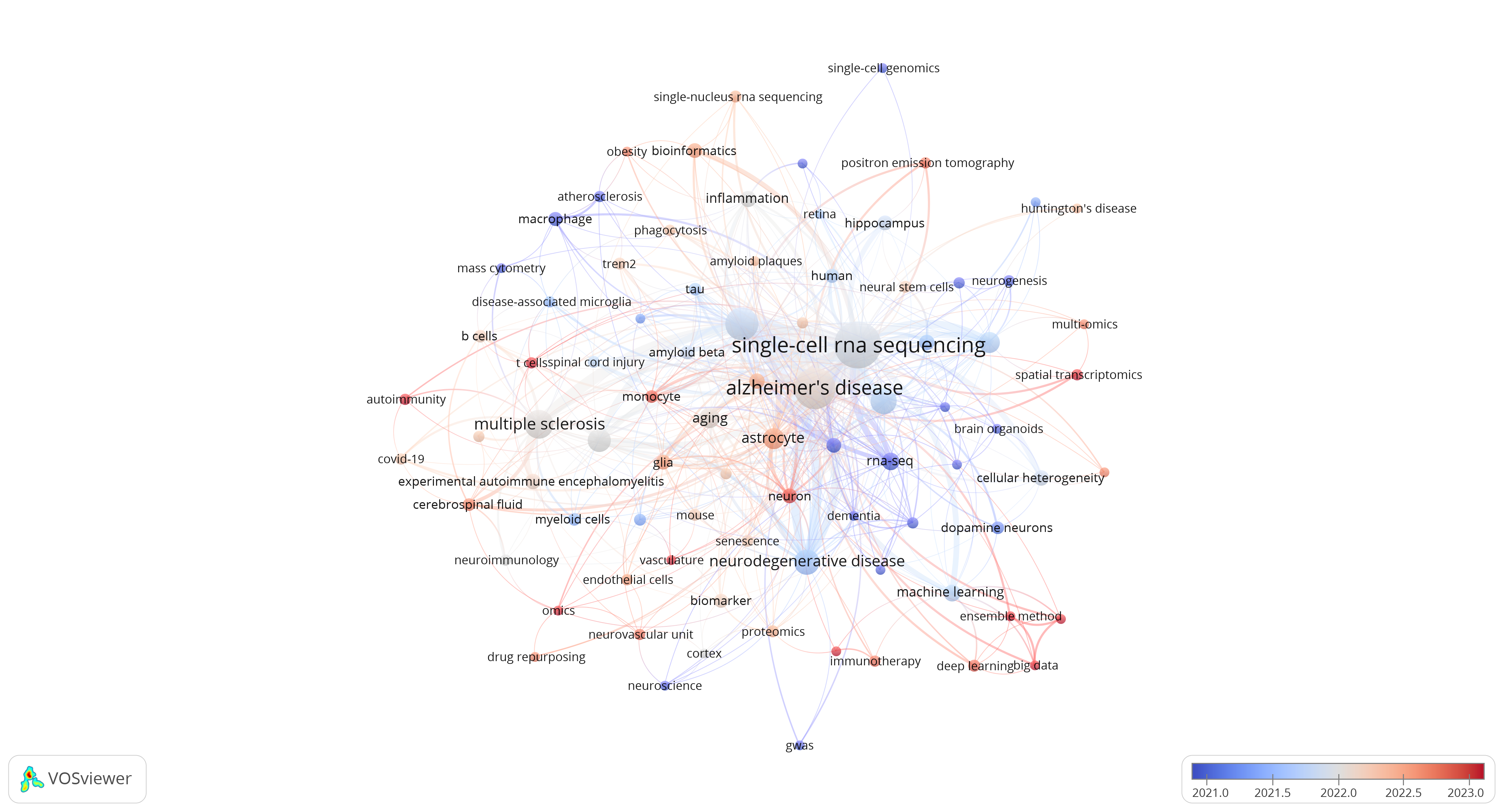

Supplement: Supplementary file 7 [file Image_6.PNG]

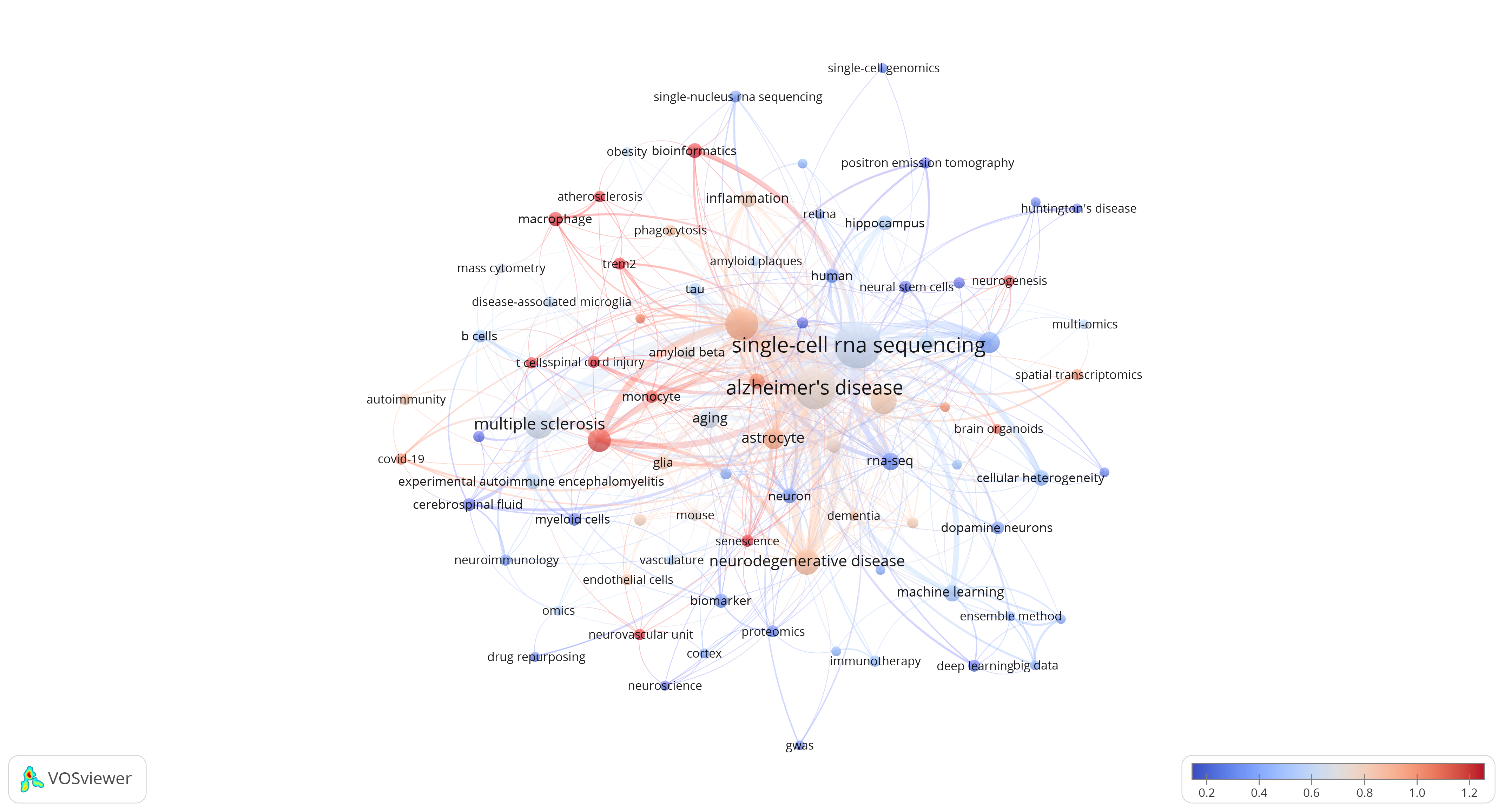

Supplement: Supplementary file 8 [file Image_7.PNG]

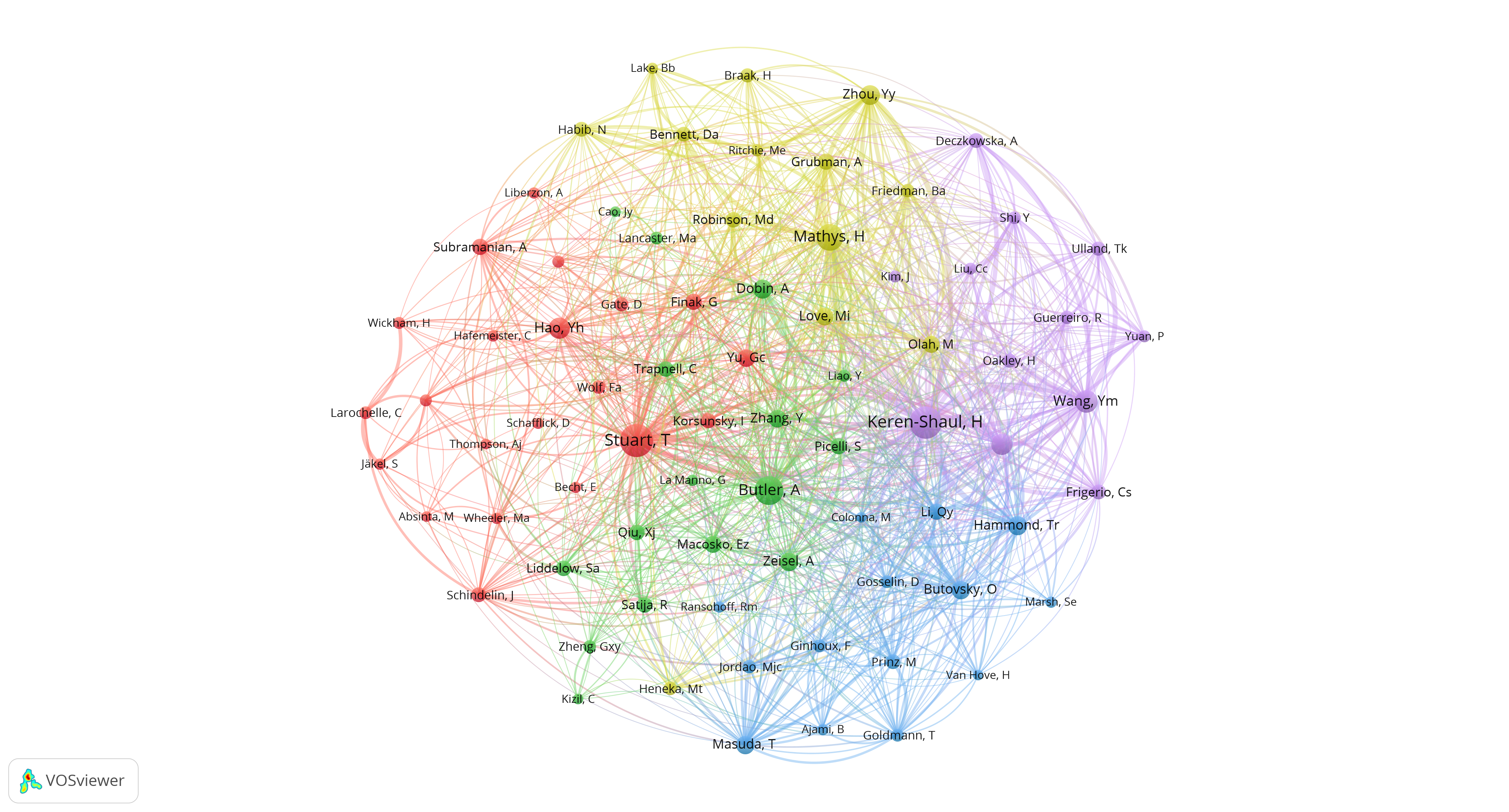

Supplement: Supplementary file 9 [file Image_8.PNG]

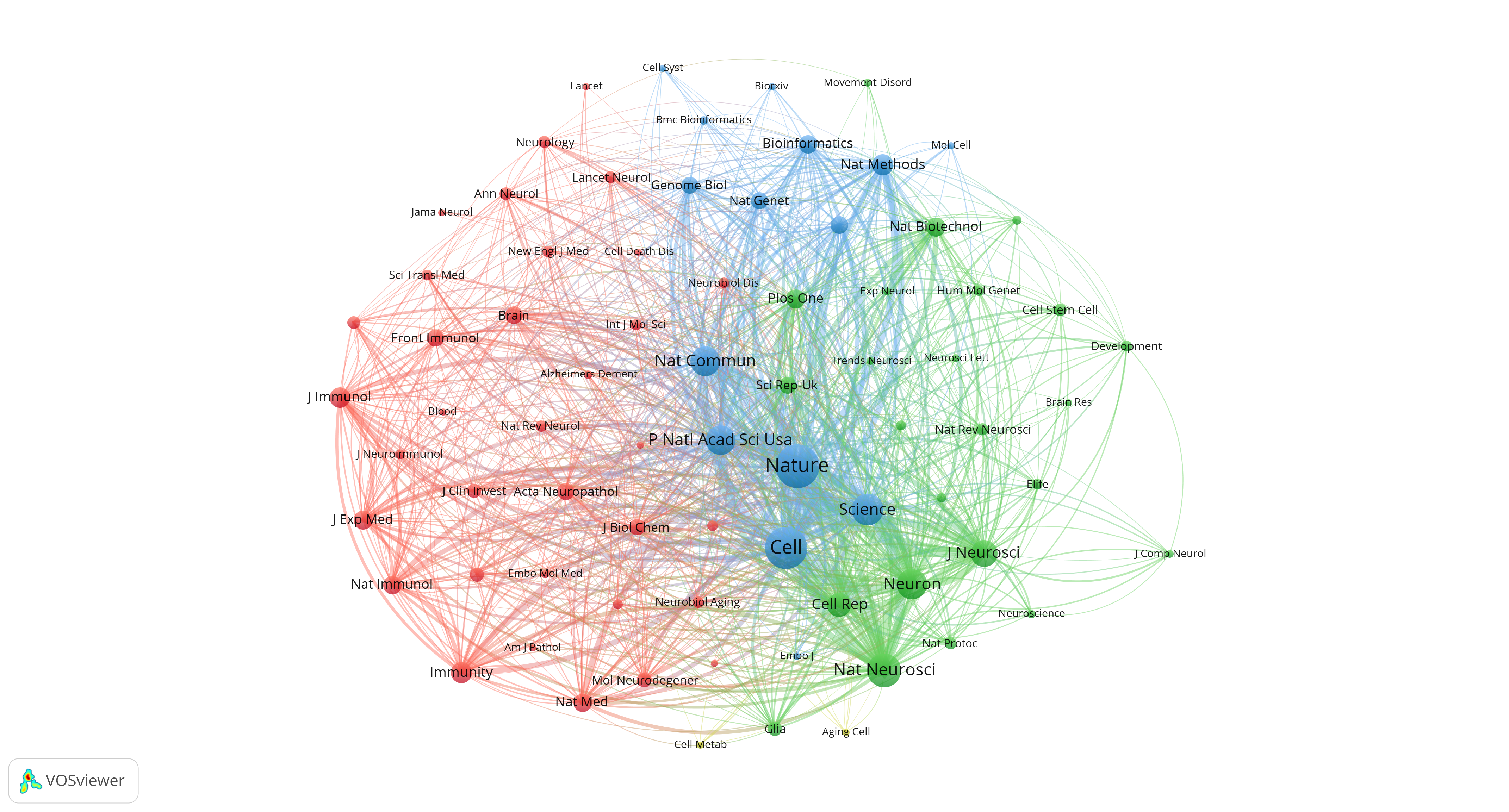

Supplement: Supplementary file 10 [file Image_9.PNG]

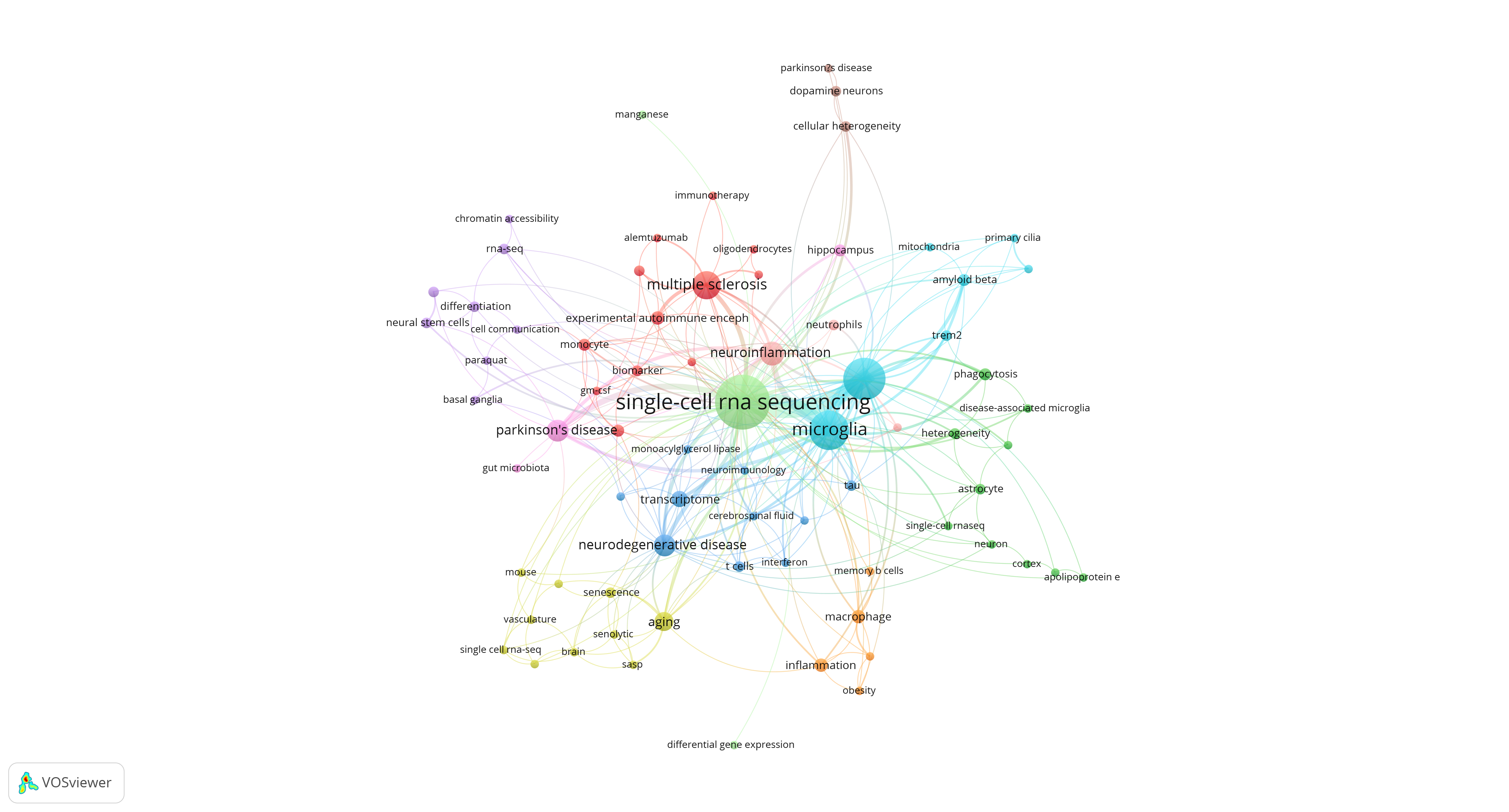

Supplement: Supplementary file 11 [file Image_10.PNG]

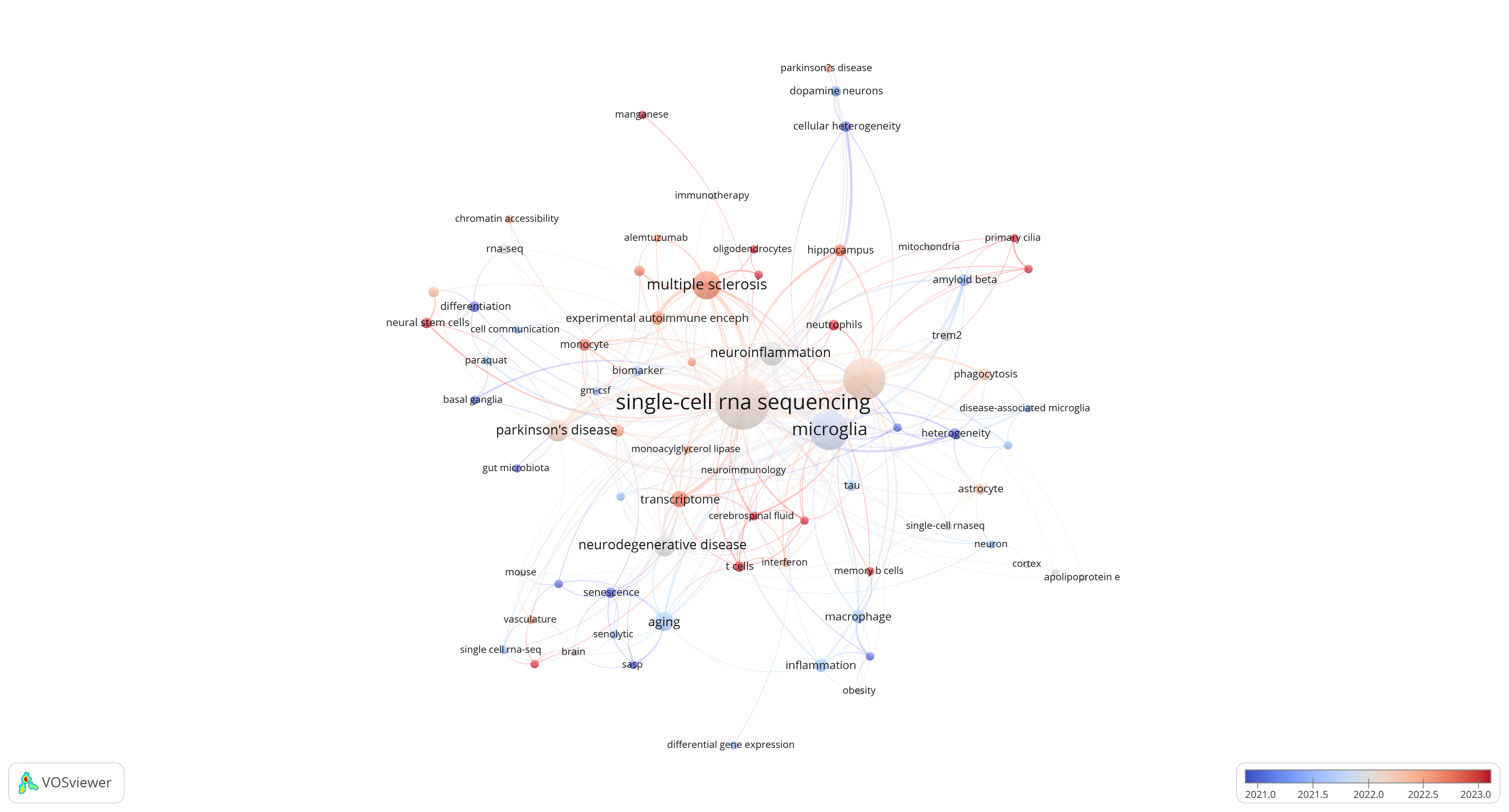

Supplement: Supplementary file 12 [file Image_11.PNG]

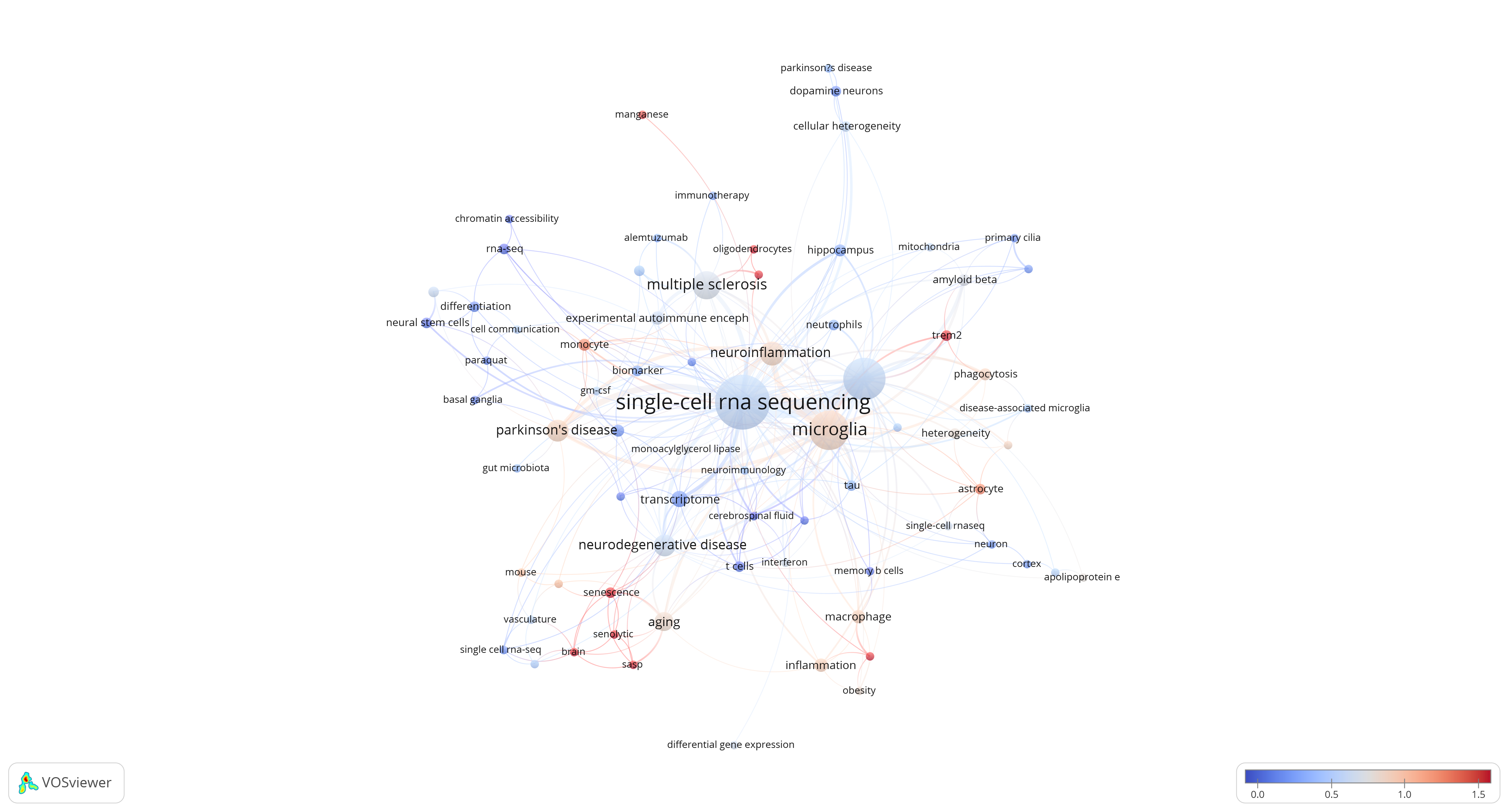

Supplement: Supplementary file 13 [file Image_12.PNG]
